# Systemic proinflammatory−profibrotic response in aortic stenosis patients with diabetes and its relationship with myocardial remodeling and clinical outcome

**DOI:** 10.1186/s12933-023-01763-1

**Published:** 2023-02-10

**Authors:** Hyun-Jung Lee, Chan Soon Park, Sahmin Lee, Jun-Bean Park, Hyung-Kwan Kim, Sung-Ji Park, Yong-Jin Kim, Seung-Pyo Lee

**Affiliations:** 1 Division of Cardiology, Department of Internal Medicine, Seoul National University Hospital, 101 Daehak-Ro, Jongno-Gu, Seoul, 03080 South Korea; 2 Division of Cardiology, Department of Internal Medicine, Asan Medical Center, University of Ulsan College of Medicine, Seoul, South Korea; 3 Department of Internal Medicine, Seoul National University College of Medicine, Seoul, South Korea; 4 Division of Cardiology, Department of Medicine, Cardiovascular Imaging Center, Heart Vascular Stroke Institute, Samsung Medical Center, Sungkyunkwan University School of Medicine, Seoul, South Korea; 5 Center for Precision Medicine, Seoul National University Hospital, Seoul, South Korea

**Keywords:** Aortic valve stenosis, Diabetes mellitus, Magnetic resonance imaging, Proteome

## Abstract

**Background:**

Previous studies have mainly focused more on how diabetes affects the valve than the myocardium in aortic stenosis (AS). In the pressure-overloaded heart, myocardial fibrosis is an important driver of the progression from compensated hypertrophy to heart failure. Using comprehensive noninvasive imaging and plasma proteomics, we investigated whether and how diabetes aggravates the remodeling of the myocardium and its relation with prognosis in AS patients.

**Methods:**

Severe AS patients were enrolled in two prospective cohorts for imaging and biomarker analysis. The imaging cohort (n = 253) underwent echocardiography and cardiac magnetic resonance, and the biomarker cohort (n = 100) blood sampling with multiplex proximity extension assay for 92 proteomic biomarkers. The composite outcome of hospitalization for heart failure admissions and death was assessed in the imaging cohort.

**Results:**

Diabetic patients were older (70.4 ± 6.8 versus 66.7 ± 10.1 years) with more advanced ventricular diastolic dysfunction and increased replacement and diffuse interstitial fibrosis (late gadolinium enhancement % 0.3 [0.0–1.6] versus 0.0 [0.0–0.5], p = 0.009; extracellular volume fraction % 27.9 [25.7–30.1] versus 26.7 [24.9–28.5], p = 0.025) in the imaging cohort. Plasma proteomics analysis of the biomarker cohort revealed that 9 proteins (E-selectin, interleukin-1 receptor type 1, interleukin-1 receptor type 2, galectin-4, intercellular adhesion molecule 2, integrin beta-2, galectin-3, growth differentiation factor 15, and cathepsin D) were significantly elevated and that pathways related to inflammatory response and extracellular matrix components were enriched in diabetic AS patients. During follow-up (median 6.3 years), there were 53 unexpected heart failure admissions or death in the imaging cohort. Diabetes was a significant predictor of heart failure and death, independent of clinical covariates and aortic valve replacement (HR 1.88, 95% CI 1.06−3.31, p = 0.030).

**Conclusions:**

Plasma proteomic analyses indicate that diabetes potentiates the systemic proinflammatory−profibrotic milieu in AS patients. These systemic biological changes underlie the increase of myocardial fibrosis, diastolic dysfunction, and worse clinical outcomes in severe AS patients with concomitant diabetes.

**Supplementary Information:**

The online version contains supplementary material available at 10.1186/s12933-023-01763-1.

## Background

Aortic stenosis (AS) is initially a disease of the heart valve but its prognosis depends greatly on the health of the myocardium. Sustained pressure overload by AS induces ventricular hypertrophy and myocardial fibrosis that lead to ventricular decompensation [1, 2]. Myocardial fibrosis is commonly observed in two forms, diffuse interstitial and focal replacement fibrosis. Both forms of fibrosis can be imaged noninvasively with cardiac magnetic resonance (CMR) with gadolinium-based contrast agents: diffuse interstitial fibrosis is quantified by extracellular volume fraction (ECV) on T1 mapping and replacement fibrosis by late gadolinium enhancement (LGE). The former is partially reversible while the latter remains even after relief of pressure overload by aortic valve replacement (AVR) in AS [3]. Both increased ECV and LGE are associated with worse prognosis in patients with AS [4–6].

‘Diabetic cardiomyopathy’ was first described from autopsies of diabetic patients who manifested with heart failure but had no evidence of coronary problems, valvular disease or hypertension [7]. Subsequent investigations demonstrated that diabetic patients have increased myocardial fibrosis explained by multiple biological and molecular mechanisms [8]. Most studies on the interaction of diabetes with AS have focused on the progression of valvular stenosis [9–11]; however, there have been only few studies that addresses the impact of diabetes on myocardial health in AS patients [12–14].

Considering that diabetes is associated with worse prognosis in AS patients [15, 16], we hypothesized that diabetes would aggravate the degree of myocardial fibrosis in AS patients. The objective of this study was two-fold; first, to elucidate the prognostic impact of diabetes in AS patients and second, to dissect its underlying mechanisms using comprehensive noninvasive imaging and plasma proteomics.

## Methods

### Study population

This study utilized two prospective cohorts of AS patients: the imaging cohort for the assessment of the myocardial health using CMR and echocardiography with long term follow-up for clinical events, and a biomarker cohort for the assessment of enriched circulating proteins using multiplex proximity extension assay.

The imaging cohort consisted of 253 patients with moderate or severe AS prospectively enrolled from 2011 to 2015 at three tertiary medical centers in Korea. All participants in this cohort underwent comprehensive echocardiography and CMR. The biomarker cohort consisted of 100 patients with severe AS undergoing surgical AVR enrolled prospectively from 2018 to 2021 at Seoul National University Hospital (Additional file 1: Fig. S1). Detailed inclusion and exclusion criteria are in Additional file 1: Additional Methods. Two separate cohorts were used in the study because patients in the imaging cohort did not undergo blood sample collection and most of the patients in the biomarker cohort did not undergo CMR and were followed for less than one year (median follow-up 6.6 [IQR 3.9–12.2] months). The study complied with the *Declaration of Helsinki*, and the cohorts were approved by the institutional review boards of each institution, with all study subjects providing written informed consent before enrollment.

Diabetic status was defined as either: (i) treatment for diabetes at enrollment with lifestyle modification, oral hypoglycemic agents, or insulin, or (ii) HbA1c ≥ 6.5% in those not previously diagnosed with diabetes and with HbA1c levels available at the period of enrollment. Medication status at enrollment was assessed for the diabetes group. Ischemic heart disease (IHD) was defined as either a history of myocardial infarction or coronary intervention, or significant coronary artery stenosis (> 50%) on angiography prior to AVR, or coronary intervention or concomitant coronary artery bypass grafting performed together with AVR.

### Echocardiographic evaluation

Echocardiography was performed using commercially available machines and the severity of AS was determined according to the contemporary guidelines [17]. The left ventricular (LV) chamber size, and systolic and diastolic function were also evaluated and categorized according to the latest guidelines [18, 19]. AS severity was defined based on aortic valve area (severe AS, aortic valve area ≤ 1 cm^2^; moderate AS, aortic valve area > 1 cm^2^ and ≤ 1.5 cm^2^) [20].

### CMR analysis

In the imaging cohort, CMR was performed at a median interval of 11 days (interquartile range 2–29 days) from the date of echocardiography. The details of the scanners, field strengths, T1 mapping sequences, contrast agents, and summary of imaging analyses for each study center are presented in Additional file 1: Table S1 [6]. Briefly, CMR scans consisted of balanced steady-state free precession cine images, pre- and post-gadolinium T1 mapping, and LGE images. The chamber sizes and myocardial mass were quantified according to a standardized protocol [5]. The degree of diffuse interstitial fibrosis was assessed by calculating ECV from the pre- and post-gadolinium T1 values measured at the short-axis mid-ventricular septum and blood pool, and hematocrit from blood samples at the time of CMR [2, 21]. Infarct-related LGE was excluded while non-infarct LGE was included in the ECV assessment [5]. The presence and extent of LGE was assessed on short-axis images acquired by phase sensitive inversion recovery sequence; the regions of LGE were drawn semi-automatically as pixels of the myocardium with a signal intensity > 5 standard deviations of the normal remote myocardium and the LGE% was calculated by dividing the LGE area by the total LV myocardial area.

### Plasma proteomics assay

Blood samples from the biomarker cohort were collected preoperatively in EDTA bottles, divided into plasma and buffy coat layers with centrifugation, and then stored at – 80 °C. For plasma proteomics analysis, deep frozen plasma samples were shipped to Olink Proteomics (Uppsala, Sweden) and the plasma levels of 92 protein biomarkers were measured using commercially available multiplex proximity extension assay kits (Cardiovascular III Panel, Additional file 1: Table S2). This high-throughput technique utilizes immunoassay with oligonucleotide-labeled antibodies followed by real-time polymerase chain reaction for simultaneous quantification of target proteins with high specificity and scalability [22]. After normalization, plasma levels were expressed for each protein in relative quantification units called normalized protein expression (NPX) using the Log_2_ scale (1 NPX difference equaling twofold change in protein concentration).

### Clinical outcomes

The clinical outcomes of interest in this study were unexpected hospitalization for heart failure that necessitated intravenous diuretics and all-cause mortality. These outcomes were assessed in the imaging cohort by review of medical records, reports from family members, and official mortality data from Statistics Korea. Patients were followed from the date of CMR to the last clinical follow-up or death.

### Statistical analysis

Continuous data are presented as mean ± standard deviation or median (interquartile range) depending on the normality of distribution, and categorical data as number (%). Characteristics were compared between the groups using the *t*-test (or Mann–Whitney test for non-normally distributed continuous variables) or the chi-square test. Comparisons of groups according to diabetes medication was conducted using the Kruskal–Wallis test. Variables associated with increased diffuse interstitial or replacement fibrosis were analyzed using logistic regression and the degree of association expressed in odds ratio (OR) with 95% confidence interval (CI). Multivariable models were constructed with the stepwise backward selection method using the Akaike information criterion or inclusion of clinically important variables such as age, sex, diabetes, hypertension, atrial fibrillation, IHD, and peak aortic velocity.

Comparison of plasma biomarker levels according to the diabetic status was performed using the Welch’s two-sample t-test, adjusting for multiple testing with the Benjamini–Hochberg method. The adjusted p-values represent the false discovery rate and p-values < 0.05 were considered significant. Logistic regression was used to assess the association of plasma biomarkers with diabetic status, adjusting for age, sex, hypertension, atrial fibrillation, IHD, and peak aortic velocity. Functional enrichment analyses were performed using g:Profiler with Gene Ontology terms.

Kaplan–Meier survival curves with log-rank tests were used to compare event-free survival according to the presence of diabetes. Cox proportional-hazards regression analyses were used to assess predictors of the endpoints and the effect size expressed as hazard ratio (HR) with 95% CI. The final multivariable model was constructed with stepwise backward selection from clinically important variables such as age, sex, diabetes, hypertension, atrial fibrillation, stroke, IHD, peak aortic velocity, LV ejection fraction by echocardiography, and AVR. Two-sided p-values < 0.05 were considered statistically significant. Analyses were conducted using R version 4.0 (Vienna, Austria) or SPSS version 25 (Chicago, USA).

## Results

### Demographic and clinical characteristics according to the presence of diabetes

There were no significant differences in the clinical characteristics and AS severity between the imaging and biomarker cohorts, except that the imaging cohort had slightly greater left atrial volume and more prevalent LV diastolic dysfunction compared to the biomarker cohort (Additional file 1: Tables S3 and S4). There was also no significant difference in the severity of diabetes assessed by diabetes medication status, HbA1c and FBS levels in the diabetic patients of the imaging and biomarker cohorts (Additional file 1: Table S4). Detailed information on the diabetes medication status of both cohorts are presented in Additional file 1: Table S5.

In the imaging cohort (n = 253), there were 66 patients with diabetes (26.1%). Among the diabetic patients, 47 (71.2%) were on oral medication only, 6 (9.1%) on insulin, and 13 (19.7%) not on any medication. Compared to non-diabetic patients, the diabetic patients were older (70.4 ± 6.8 vs. 66.7 ± 10.1 years, p = 0.001), had a higher prevalence of hypertension (72.7% vs. 55.1%, p = 0.018) and IHD (28.8% vs. 9.1%, p < 0.001), and tended to use more diuretics (Table 1).

**Table 1:** Demographic and clinical characteristics of the patients in the imaging cohort and biomarker cohort

	Imaging cohort				Biomarker cohort			
	Total (n = 253)	Non-DM (N = 187)	DM (N = 66)	p-value	Total (n = 100)	Non-DM (N = 73)	DM (N = 27)	p-value
Age (years)	67.7 ± 9.5	66.7 ± 10.1	70.4 ± 6.8	0.001	66.6 ± 9.6	65.5 ± 9.9	69.5 ± 8.2	0.064
Male	127 (50.2)	93 (49.7)	34 (51.5)	0.916	60 (60.0)	43 (58.9)	17 (63.0)	0.890
Hypertension	151 (59.7)	103 (55.1)	48 (72.7)	0.018	58 (58.0)	39 (53.4)	19 (70.4)	0.195
Atrial fibrillation	31 (12.3)	18 (9.6)	13 (19.7)	0.054	13 (13.0)	11 (15.1)	2 (7.4)	0.499
Stroke	21 (8.3)	13 (7.0)	8 (12.1)	0.294	11 (11.0)	6 (8.2)	5 (18.5)	0.271
Ischemic heart disease	56 (22.1)	31 (16.6)	25 (37.9)	0.001	19 (19.0)	10 (13.7)	9 (33.3)	0.053
Creatinine (mg/dL)	0.88 ± 0.23	0.86 ± 0.20	0.91 ± 0.30	0.180	1.12 ± 1.37	1.07 ± 1.19	1.25 ± 1.80	0.635
Euroscore II	1.6 ± 1.5	1.3 ± 0.7	2.5 ± 2.5	< 0.001	1.7 ± 1.8	1.7 ± 1.5	1.8 ± 2.3	0.717
NYHA III-IV	55 (21.8)	36 (19.4)	19 (28.8)	0.155	16 (16.0)	13 (17.8)	3 (11.1)	0.614
*Medication*								
ACE inhibitor/ARB	111 (43.9)	75 (40.1)	36 (54.5)	0.059	37 (37.0)	25 (34.2)	12 (44.4)	0.481
Beta-blocker	116 (45.8)	88 (47.1)	28 (42.4)	0.613	48 (48.0)	33 (45.2)	15 (55.6)	0.487
Calcium channel blocker	56 (22.1)	43 (23.0)	13 (19.7)	0.702	–	–	–	
Diuretics	99 (39.1)	64 (34.2)	35 (53.0)	0.011	–	–	–	
*Diabetes medication*								
None			13 (19.7)				4 (14.8)	
Oral medication only			47 (71.2)				19 (70.4)	
Insulin user			6 (9.1)				4 (14.8)	
HbA1c		5.7 [5.5–5.9] (n = 103)	6.6 [6.2–7.5] (n = 58)	< 0.001		5.8 [5.5–6.0] (n = 64)	7.0 [6.4–7.4]	< 0.001
FBS		109 [96–128] (n = 85)	132 [110–172] (n = 52)	< 0.001			120 [107–147] (n = 25)	

ACE, angiotensin converting enzyme; ARB, angiotensin receptor blocker; DM, diabetes mellitus; FBS, fasting blood sugar; NYHA, New York Heart Association

In the biomarker cohort (n = 100), there were 27 patients with diabetes (27%), of whom 19 (70.4%) were on oral medication only, 4 (14.8%) on insulin, and 4 (14.8%) on no medication (Table 1). There were no statistical differences in the clinical or demographic parameters between the diabetic and non-diabetic patients in the biomarker cohort. White blood cell count or neutrophil/lymphocyte ratio was also not different between the diabetic and non-diabetic patients (Additional file 1: Table S6).

### Increased risk of myocardial fibrosis on noninvasive imaging in diabetic AS patients

**Fig. 1:**
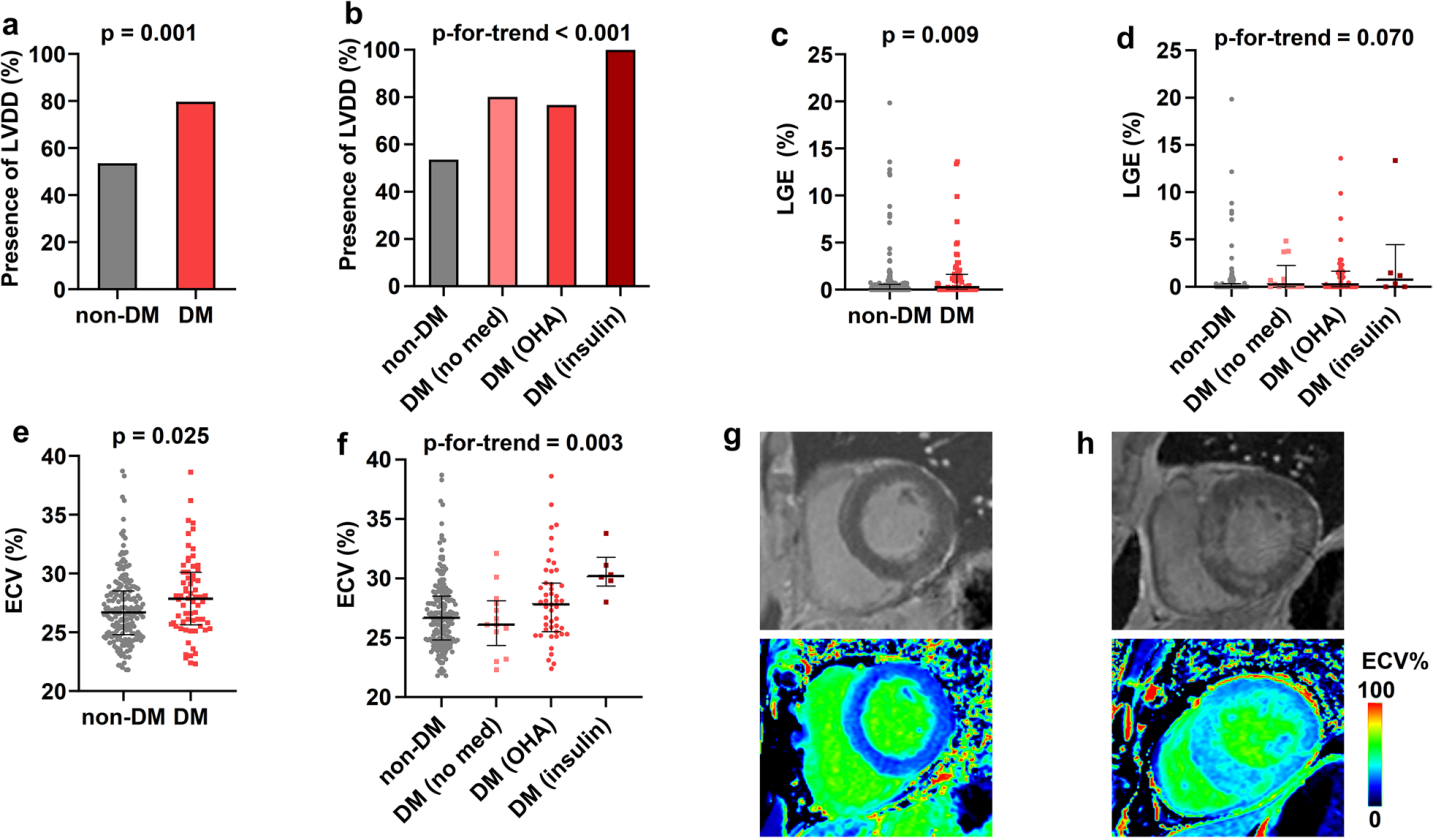
Myocardial fibrosis and left ventricular diastolic function in aortic stenosis patients according to diabetes and diabetes medication status. **a**, **b** Comparison of the degree of diastolic dysfunction in **a** patients with versus without diabetes and in **b** patients stratified by the diabetes medication status. **c**, **d** Comparison of late gadolinium enhancement (LGE) in **c** patients with versus without diabetes and in **d** patients stratified by the diabetes medication status. **e**, **f** Comparison of extracellular volume fraction (ECV) in **e** patients with versus without diabetes and in **f** patients stratified by the diabetes medication status. **g**, **h** Representative LGE and ECV images of cardiac magnetic resonance taken from **g** a non-diabetic aortic stenosis patient (no LGE; ECV 26.4%) versus **h** a diabetic aortic stenosis patient on oral hypoglycemic agent (midwall LGE in the septal and lateral wall; ECV 34.5%). DM, diabetes mellitus; ECV, extracellular volume; LGE, late gadolinium enhancement; LVDD, left ventricular diastolic dysfunction; OHA, oral hypoglycemic agents

There was no significant difference in AS severity, aortic valve area and mean pressure gradient between diabetic and non-diabetic patients in the imaging cohort, although the peak aortic velocity was slightly lower in patients with diabetes (4.5 ± 0.9 vs. 4.8 ± 8.0 m/s, p = 0.036). However, compared to the non-diabetic patients, the diabetic patients had worse LV diastolic function (prevalence of LV diastolic dysfunction 79.7% vs. 53.5%, p = 0.001) (Fig. 1A), supported by lower e′ velocity, higher E/e′ and tricuspid regurgitation peak velocity, and a shorter mitral deceleration time (Table 2). Notably, the prevalence of grade 2 or 3 LV diastolic dysfunction was higher in the diabetic patients. When stratified by diabetes medication as a surrogate marker of chronicity and severity of diabetes, there was also a higher prevalence of LV diastolic dysfunction with needs for more intensive diabetes treatment (Fig. 1B, p-for-trend < 0.001). In the biomarker cohort as well, while there was no significant difference in AS severity, the diabetic patients had lower e′ velocity with a tendency towards more advanced LV diastolic dysfunction than the nondiabetic patients (Additional file 1: Table S7).

**Table 2:** Echocardiography and cardiac magnetic resonance analysis of the patients with and without diabetes in the imaging cohort

	Total (n = 253)	Non-DM (N = 187)	DM (N = 66)	p-value
*Echocardiography*				
LV end-diastolic dimension (mm)	50.2 ± 6.7	50.1 ± 6.8	50.6 ± 6.4	0.633
LV end-systolic dimension (mm)	31.8 ± 7.8	31.5 ± 7.4	32.4 ± 9.0	0.423
LV mass index (g/m^2^)	132 ± 40	133 ± 41	130 ± 37	0.625
Relative wall thickness	0.44 ± 0.09	0.44 ± 0.09	0.44 ± 0.09	0.974
LV ejection fraction (%)	59.6 ± 9.9	60.3 ± 8.9	57.6 ± 12.0	0.104
Left atrial diameter (mm)	43.8 ± 6.9	43.4 ± 7.1	44.8 ± 6.2	0.181
E velocity (m/s)	0.79 ± 0.38	0.76 ± 0.36	0.88 ± 0.43	0.038
A velocity (m/s)	0.87 ± 0.29	0.86 ± 0.30	0.90 ± 0.26	0.335
Deceleration time (ms)	247 ± 79	253 ± 83	229 ± 64	0.022
E/A	0.78 [0.63–1.10]	0.78 [0.63–1.09]	0.78 [0.60–1.10]	0.894
e′ velocity (cm/s)	4.6 ± 1.4	4.7 ± 1.4	4.2 ± 1.4	0.007
a′ velocity (cm/s)	7.3 ± 1.8	7.3 ± 1.7	7.2 ± 2.1	0.714
s′ velocity (cm/s)	5.1 ± 1.4	5.2 ± 1.4	4.9 ± 1.5	0.132
E/e′	15.7 [12.3–21.2]	15.2 [12.1–20.0]	19.2 [13.3–31.0]	0.002
TR Vmax (m/s)	2.5 ± 0.4	2.5 ± 0.4	2.7 ± 0.6	0.013
PASP (mmHg)	34.4 ± 9.4	33.3 ± 8.0	38.0 ± 12.4	0.015
Left atrial volume index (mL/m^2^)	49.2 [39.0–62.7]	50.8 [38.0–63.2]	48.0 [41.2–58.0]	0.642
Peak aortic velocity (m/s)	4.7 ± 0.8	4.8 ± 0.8	4.5 ± 0.9	0.036
AV mean PG (mmHg)	55 ± 21	56 ± 22	51 ± 21	0.095
AV area (cm^2^)	0.76 ± 0.23	0.76 ± 0.22	0.74 ± 0.25	0.371
AS severity (by AV area)				0.803
Severe AS	218 (86.5)	162 (87.1)	56 (84.8)	
Moderate AS	34 (13.5)	24 (12.9)	10 (15.2)	
Presence of LVDD (n = 231)	139 (60.2)	92 (53.5)	47 (79.7)	0.001
LVDD grade (n = 208)				0.013
Normal	40 (19.2)	38 (23.9)	2 (4.1)	
Indeterminate	52 (25.0)	42 (26.4)	10 (20.4)	
Grade 1 LVDD	21 (10.1)	14 (8.8)	7 (14.3)	
Grade 2 LVDD	85 (40.9)	58 (36.5)	27 (55.1)	
Grade 3 LVDD	10 (4.8)	7 (4.4)	3 (6.1)	
*Cardiac magnetic resonance*				
Indexed LVEDV (mL/m^2^)	109.3 ± 47.8	112.2 ± 50.8	100.9 ± 36.8	0.056
Indexed LVESV (mL/m^2^)	45.1 ± 34.3	45.6 ± 35.9	43.8 ± 29.3	0.715
Indexed LVSV (mL/m^2^)	58.0 ± 17.4	60.5 ± 18.0	50.9 ± 13.2	< 0.001
LV ejection fraction (%)	62.9 ± 13.8	64.0 ± 13.5	59.5 ± 14.0	0.021
LV mass index (g/m^2^)	101.6 ± 36.7	101.6 ± 37.0	101.3 ± 36.1	0.950
LV mass/volume ratio^a^ (g/mL)	1.00 ± 0.34	0.98 ± 0.35	1.05 ± 0.32	0.191
ECV (%)	26.8 [25.1–28.9]	26.7 [24.9–28.5]	27.9 [25.7–30.1]	0.025
Presence of LGE	112 (44.3)	75 (40.1)	37 (56.1)	0.036
LGE (%)	0.0 [0.0–0.7]	0.0 [0.0–0.5]	0.3 [0.0–1.6]	0.009
LGE (%) in patients with LGE	0.8 [0.2–1.9]	0.6 [0.2–1.5]	1.2 [0.4–2.9]	0.026

AV, aortic valve; ECV, extracellular volume fraction; LGE, late gadolinium enhancement; LV, left ventricular; LVDD, LV diastolic dysfunction; LVEDV, LV end-diastolic volume; LVESV, LV end-systolic volume; LVSV, LV stroke volume; PASP, pulmonary artery systolic pressure; PG, pressure gradient; TR, tricuspid regurgitation; Vmax, maximum velocity

^a^LV mass divided by LV end-diastolic volume

On analysis of CMR in the participants of the imaging cohort, a significant increase of replacement fibrosis was observed in diabetic patients (Table 2). The LGE was present in 56% of diabetic patients compared to 40% in non-diabetic patients (p = 0.036), and LGE was more frequently present in patients with LGE with need for more intensive diabetes treatment (p-for-trend = 0.016) (Additional file 1: Fig. S2). The extent of LGE was also higher in the diabetic patients (Fig. 1C, LGE% in the entire population 0.3 [0.0–1.6] vs. 0.0 [0.0–0.5], p = 0.009) (LGE% in those with any LGE 1.2 [0.4–2.9] vs. 0.6 [0.2–1.5], p = 0.026). There was also a tendency for higher LGE% with more intensive diabetes treatment (p-for-trend = 0.070) (Fig. 1D, Additional file 1: Table S8). As for the degree of diffuse interstitial fibrosis, the ECV was higher in patients with diabetes (Fig. 1E, ECV% 27.9 [25.7–30.1] vs. 26.7 [24.9–28.5], p = 0.025). Similar to the analysis of LGE%, the ECV% was significantly higher with more intensive treatment of diabetes (p-for-trend = 0.003) (Fig. 1F, Additional file 1: Table S8). Exploratory analyses in the subsets of patients with HbA1c and FBS levels available (n = 162 and n = 137, respectively) also showed that the presence of LV diastolic dysfunction and parameters of fibrosis increased numerically with worse glycemic control, albeit with variable statistical significance (Additional file 1: Tables S9 and S10). The LV stroke volume and ejection fraction measured by CMR were lower in diabetic patients.

Diabetes was significantly associated with increased diffuse interstitial and replacement fibrosis on univariable and multivariable analyses (Table 3). The presence of diabetes showed the strongest associations with the highest quartile of both increased interstitial or replacement fibrosis, i.e. ECV% (adjusted HR 1.9, p-value = 0.05) and LGE% (adjusted HR 3.0, p-value < 0.01).

**Table 3:** Predictors of increased diffuse interstitial or replacement myocardial fibrosis (highest quartile)

Diffuse myocardial fibrosis (ECV)						
	Univariable		Multivariable model 1^a^		Multivariable model 2^b^	
	Crude HR	p-value	Adjusted HR	p-value	Adjusted HR	p-value
Age (years)	1.00 (0.97–1.03)	0.772	0.98 (0.95–1.01)	0.234		
Male	1.18 (0.66–2.11)	0.578	1.11 (0.61–2.04)	0.730		
Diabetes	2.17 (1.17–4.04)	0.015	1.94 (0.99–3.79)	0.052	1.90 (1.00–3.61)	0.049
Hypertension	1.48 (0.80–2.71)	0.208	1.29 (0.66–2.51)	0.456	1.88 (0.96–3.68)	0.064
Atrial fibrillation	0.93 (0.38–2.28)	0.874	0.82 (0.32–2.08)	0.675		
Ischemic heart disease	2.18 (1.14–4.17)	0.018	1.82 (0.90–3.66)	0.094		
Peak aortic velocity (m/s)	0.76 (0.54–1.09)	0.139	0.83 (0.57–1.19)	0.311		

Replacement fibrosis (LGE)						
	Univariable		Multivariable model 1^a^		Multivariable model 2^b^	
	Crude HR	p-value	Adjusted HR	p-value	Adjusted HR	p-value
Age (years)	1.03 (0.99–1.06)	0.100	1.01 (0.98–1.05)	0.427		
Male	2.17 (1.20–3.93)	0.010	2.14 (1.14–4.01)	0.018	2.10 (1.13–3.92)	0.019
Diabetes	3.32 (1.80–6.12)	< 0.001	2.96 (1.53–5.73)	0.001	3.04 (1.60–5.76)	< 0.001
Hypertension	1.44 (0.79–2.62)	0.235	1.18 (0.60–2.30)	0.631		
Atrial fibrillation	1.30 (0.57–3.01)	0.533	1.09 (0.45–2.68)	0.848		
Ischemic heart disease	2.54 (1.34–4.81)	0.004	1.78 (0.89–3.56)	0.105	1.81 (0.92–3.58)	0.085
Peak aortic velocity (m/s)	1.03 (0.73–1.45)	0.869	1.21 (0.83–1.76)	0.312		

Important clinical variables are shown in the first column

ECV, extracellular volume fraction; HR, hazard ratio; LGE, late gadolinium enhancement

^a^Model 1 was adjusted for all variables in the first column

^b^Model 2 was constructed with stepwise backward selection from variables presented in the first column

### Upregulation of the proinflammatory−profibrotic pathways in the plasma proteome of diabetic AS patients

**Fig. 2:**
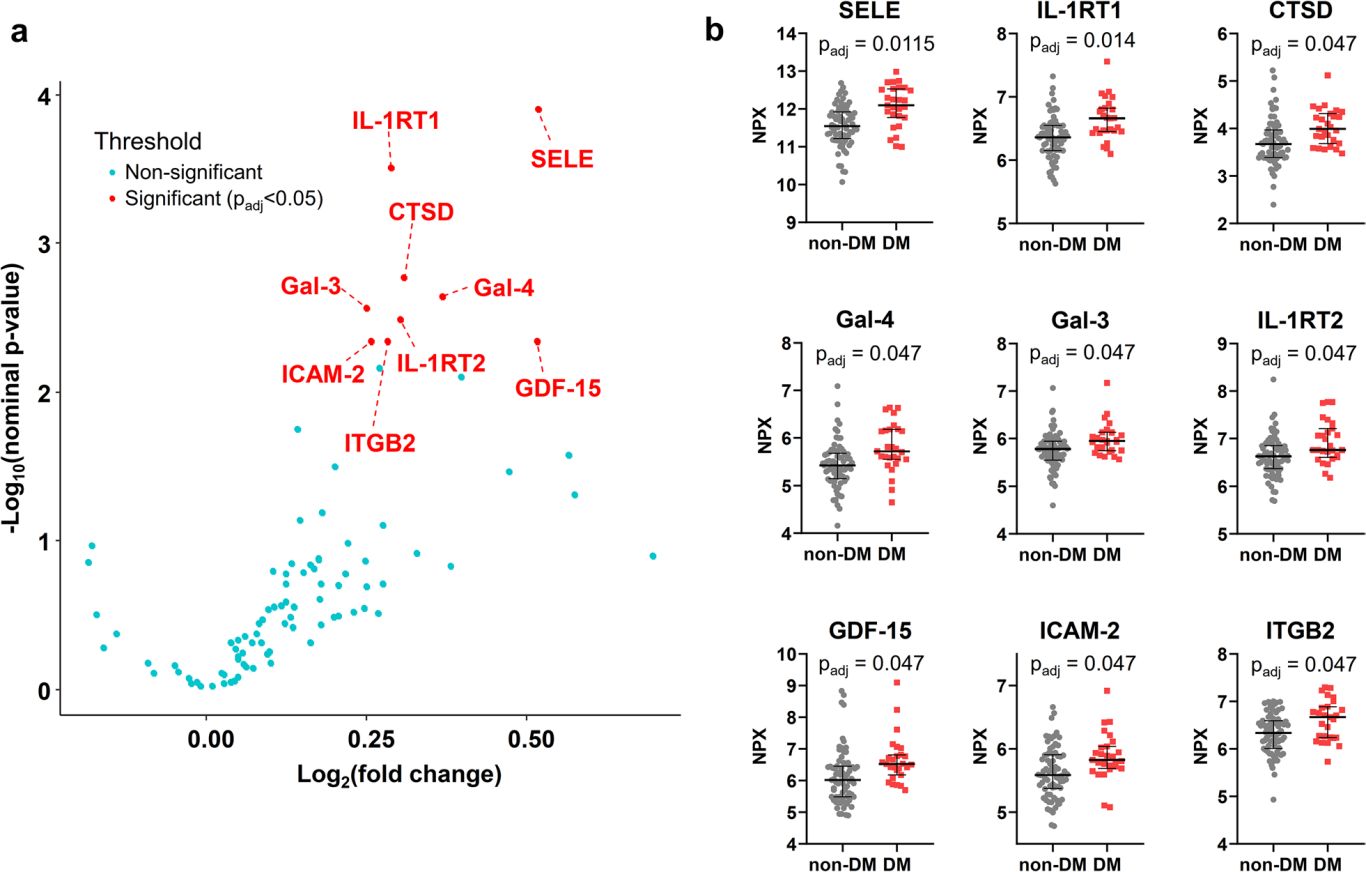
Significantly upregulated plasma proteins in AS patients with diabetes on proteomic analysis. **a** Volcano plot identifying the significantly upregulated proteins (annotated in red) in the plasma proteome of diabetic AS patients. **b** Scatter plots comparing the plasma levels of differentially expressed proteins in AS patients with and without diabetes. All comparisons were adjusted for multiple testing with the Benjamini & Hochberg method, with adjusted p-values representing the false discovery rate. The horizontal bars for each group indicate median and interquartile range. adj, adjusted; AS, aortic stenosis; DM, diabetes mellitus; SELE, E-selectin; IL-1RT1, interleukin-1 receptor type 1; CTSD, cathepsin D; Gal-4, galectin-4; Gal-3, galectin-3; IL-1RT2, interleukin-1 receptor type 2; GDF-15, growth differentiation factor 15; ICAM-2, intercellular adhesion molecule 2; ITGB2, integrin beta-2

**Fig. 3:**
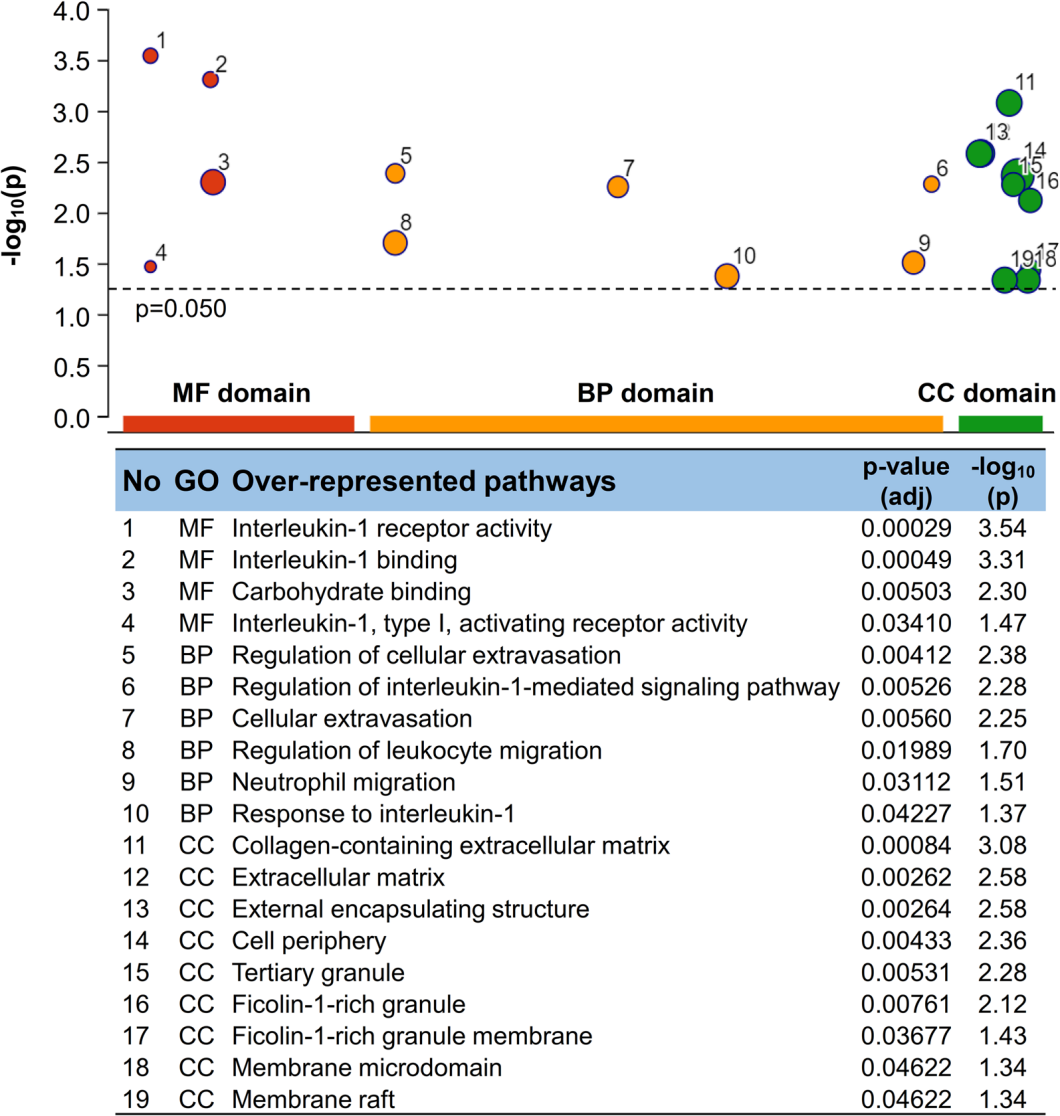
Over-represented pathways in the plasma proteome of AS patients with diabetes. Functional enrichment analyses using the g:Profiler with Gene Ontology terms were performed with the proteomic analysis results and are presented in a Manhattan plot. The sizes of circles signify intersection size. adj, adjusted; AS, aortic stenosis; BP, biological process; CC, cellular component; GO, Gene Ontology domains; MF, molecular function

The distribution of NPX values for each sample are shown in Additional file 1: Fig. S3. Among the 92 candidate proteins in the plasma proteomics analysis of the biomarker cohort, 9 proteins (E-selectin, interleukin-1 receptor type 1, interleukin-1 receptor type 2, galectin-4, intercellular adhesion molecule 2, integrin beta-2, galectin-3, growth differentiation factor 15 [GDF-15], and cathepsin D) were significantly upregulated in diabetic AS patients compared to non-diabetic AS patients (Fig. 2, Additional file 1: Table S11) (false discovery rate < 5% and minimal fold change of 1.15 [Log_2_(fold change) > 0.20])[23]. There were no proteins that were significantly downregulated in diabetic AS patients. These proteins biomarkers were independently associated with diabetic status after adjustment for age, sex, atrial fibrillation, IHD, peak aortic velocity, and LV ejection fraction, with odds ratios ranging from 3 to 13 per twofold increase in each protein level (Additional file 1: Table S12). Biomarker levels also did not differ significantly in diabetic patients according to metformin use (Additional file 1: Table S13). Pathway over-representation analyses of the upregulated plasma proteome indicated that pathways related to proinflammatory response and extracellular matrix components were enriched in the plasma of AS patients with concomitant diabetes (Fig. 3, Additional file 1: Table S14).

Exploratory pathway over-representation analyses with plasma biomarkers fulfilling a more lenient criteria of nominal p-value < 0.050 and minimal fold change of 1.15 showed similar results. A total of 16 biomarkers met this criteria (interleukin-18-binding protein, C–C motif chemokine 16, tissue-type plasminogen activator, scavenger receptor cysteine-rich type 1 protein M130, pulmonary surfactant-associated protein D, insulin-like growth factor-binding protein 1, and urokinase plasminogen activator surface receptor as well as the aforementioned 9 proteins; Additional file 1: Fig. S4A) and the tendency for enrichment of proinflammatory and profibrotic pathways was persistent (Additional file 1: Fig. S4B, Table S15).

### Clinical outcomes according to the presence of diabetes

The participants in the imaging cohort were followed for a median 6.3 (interquartile range 5.2–7.2) years and nearly all patients (n = 232, 91.7%) received AVR during follow-up. Most patients received surgical AVR (n = 216, 93.1%). The proportion of patients receiving AVR (92.0% vs 90.9%, p = 0.991) or the type of AVR, either surgical or transcatheter (surgical AVR 94.8% vs 88.3%, p = 0.162), was not significantly different between the non-diabetic and diabetic AS patients. There were 53 events of unexpected admission for heart failure or death (20.9%) (Additional file 1: Table S16).

**Fig. 4:**
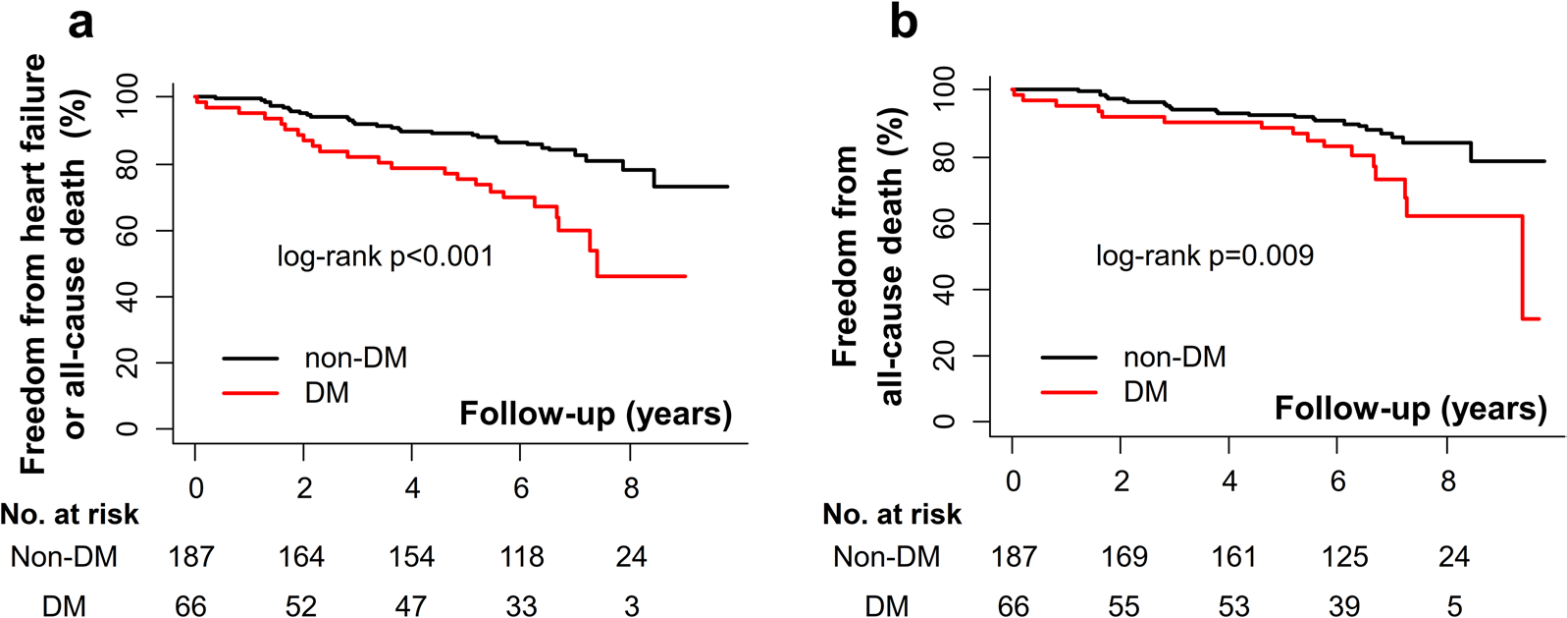
Comparison of event-free survival in AS patients according to diabetes. Kaplan–Meier analysis with p-values by the log-rank test are presented for **a** the composite outcome of admission for heart failure and all-cause death, and **b** all-cause death. AS, aortic stenosis; DM, diabetes mellitus

The incidence of the composite clinical events was significantly higher in diabetic AS patients compared to the non-diabetic AS subjects (Fig. 4A); all-cause mortality was also higher in diabetic AS subjects (Fig. 4B). Diabetes was a significant predictor of heart failure and all-cause death (HR 2.09, 95% CI 1.20–3.63, p = 0.009), independent of age, sex, atrial fibrillation, IHD, LV ejection fraction, and AVR (Table 4).

**Table 4:** Predictors of unexpected admission for heart failure or all-cause mortality

	Univariable		Multivariable model^a^	
	Crude HR	p-value	Adjusted HR	p-value
Age (years)	1.07 (1.03–1.11)	< 0.001	1.06 (1.02–1.11)	0.002
Male	1.67 (0.96–2.89)	0.069	1.56 (0.88–2.75)	0.130
Diabetes	2.71 (1.57–4.68)	< 0.001	2.09 (1.20–3.63)	0.009
Hypertension	1.57 (0.88–2.80)	0.125		
Atrial fibrillation	2.88 (1.56–5.30)	0.001	2.77 (1.47–5.23)	0.002
Stroke	1.39 (0.60–3.26)	0.445		
Ischemic heart disease	2.73 (1.57–4.74)	< 0.001	1.91 (1.09–3.37)	0.025
Peak aortic velocity (m/s)	0.65 (0.47–0.89)	0.008		
AV replacement	0.33 (0.17–0.64)	0.001	0.22 (0.11–0.46)	< 0.001
LV ejection fraction (%)	0.97 (0.95–0.99)	0.011	0.97 (0.95–0.99)	0.010

AV, aortic valve; HR, hazard ratio; LV, left ventricular

^a^Constructed with stepwise backward selection from variables presented in the first column

In the analysis of patients who underwent AVR (n = 232), the AS patients with concomitant diabetes also had worse clinical outcomes (Additional file 1: Fig. S5, Table S17), again suggesting that diabetes has a pervasive systemic effect on the myocardial health even after relief of pressure overload by AVR. Diabetes was associated with clinical events independently of the type of AVR and other clinical variables (Additional file 1: Table S18).

## Discussion

Herein, we demonstrated that AS patients with diabetes compared to non-diabetic patients had increased diffuse interstitial and replacement fibrosis by CMR analysis of the myocardium. An in-depth investigation of plasma proteomics demonstrated that factors related to proinflammatory response and extracellular matrix components were enriched in diabetic AS patients. These diabetic AS patients had a significantly higher incidence of heart failure and death than the non-diabetic patients. These results suggest that diabetes is associated with effects that potentiate systemic proinflammatory and profibrotic milieu in the pressure-overloaded myocardium, which translates into worse clinical outcomes even after AVR.

Diabetes mellitus is a systemic disease that affects the myocardium directly [8]. Patients with otherwise uncomplicated diabetes show reduced cardiopulmonary performance [24, 25], which is associated with increased myocardial fibrosis [26]. Microvascular function is also impaired in diabetes [27], and therefore, the myocardium may be more vulnerable to ischemic insult [28]. Studies utilizing CMR in the general population have suggested that diffuse interstitial and replacement fibrosis are increased in diabetic compared to non-diabetic subjects, and both forms of fibrosis are associated with heart failure and mortality events [29–31].

There have been few studies on how diabetes impacts myocardial remodeling in AS. We found that the degree of myocardial fibrosis and diastolic dysfunction is significantly more advanced in diabetic AS patients. An invasive histological study using myocardial biopsies in 60 AS patients undergoing AVR suggested that patients with concomitant AS and diabetes had more myocardial fibrosis and higher cardiomyocyte stiffness [13]. Using noninvasive imaging to examine the entire myocardium, we show that the histologic evidence from the previous study [13] holds true in a larger AS population.

In AS patients, diabetes is independently associated with an increase in both mid-term and long-term mortality in those undergoing AVR, as well as in those with asymptomatic AS patients managed conservatively [15, 16, 32]. Herein, we suggest the missing link between diabetes and outcome in AS by demonstrating the association between diabetes and myocardial fibrosis. Moreover, the need for more intensive diabetes treatment as a marker of diabetes severity was associated with a greater degree of myocardial fibrosis and LV diastolic dysfunction, especially in insulin-treated diabetic patients. Previous studies have also shown that the prognosis of diabetic AS patients even after AVR are significantly worse especially in those treated with insulin [16, 32]. This suggests that factors other than the stenotic valve in AS is responsible for the worse prognosis in diabetic AS patients and that relief of the stenotic valve may not be enough for optimal outcome.

In the pressure-overloaded heart, myocardial fibrosis is an important driver of the progression from compensated hypertrophy to heart failure with diastolic and systolic dysfunction, findings demonstrated in both histological and imaging studies [1, 33, 34]. In the current study, AS patients with diabetes had greater replacement and diffuse interstitial fibrosis compared to non-diabetic counterparts. Although the difference in both measures of fibrosis between the diabetics versus non-diabetics may seem small, this difference is clinically significant as shown by previous investigations demonstrating 11% and 10% higher mortality with each 1% increase of %LGE and %ECV, respectively [4, 5]. Furthermore, there is a characteristic non-linear and threshold effect of %LGE and %ECV on outcome in AS [6], suggesting that even a small increase of fibrosis can lead to significantly worse outcomes. Diabetes is also associated with poor LV mass regression after AVR [14], suggesting that it continues to affect myocardial remodeling after relief of pressure overload.

This leads us to question whether and how diabetes changes the systemic milieu and ultimately, the myocardium. Circulating protein biomarkers provide important clues to pathophysiological mechanisms of diseases, and high-throughput proteomic methods can measure a multitude of proteins simultaneously. In previous plasma proteome studies of AS patients, higher GDF-15 was associated with poor LV reverse-remodeling and increased mortality after AVR [35, 36]. In plasma proteome analysis of patients with heart failure, diabetic patients had higher circulating GDF-15 and galectin-4 levels [23], and over-representation of pathways related to inflammation, cardiac remodeling, and fibrosis [23, 37]. We found that E-selectin, interleukin-1 receptor type 1, interleukin-1 receptor type 2, galectin-3, galectin-4, intercellular adhesion molecule 2, integrin beta-2, GDF-15, and cathepsin D levels were significantly upregulated in diabetic AS patients, proteins which have been implicated in inflammation, cardiac fibrosis and remodeling, atherosclerosis, and heart failure [38–44]. Furthermore, over-representation analyses of the plasma proteome demonstrated that pathways related to neutrophil activation, interleukin-1 and amplification of inflammation, leukocyte migration, and extracellular matrix were enriched in diabetic AS patients. Our study supports that upregulation of signals related to inflammation and extracellular matrix expansion are important pathophysiological processes systemically mediated by diabetes in AS patients, which in turn, may aggravate the degree of myocardial fibrosis and lead to worse outcomes.

Our study suggests that clinicians should be aware of the significantly higher clinical events in AS patients with diabetes. According to our analysis, diabetes not only damages the stenotic valve [9–11], but also, the health of the myocardium, with its effect pervading even after AVR. In particular, diabetes patients treated with insulin may be at the highest risk. Our findings suggest that the changes in the myocardium by AS are already more advanced in diabetes patients and it can be assumed that the systemic proinflammatory−profibrotic milieu will continue with diabetes even after AVR. This also suggests that AVR may not be the final treatment for AS when the patient has diabetes. Considering the systemic proinflammatory−profibrotic environment by diabetes, our findings call for further studies on whether anti-inflammatory approaches may be beneficial in AS patients with concomitant diabetes, to prevent adverse myocardial remodeling and to improve clinical outcomes. Exciting options testing this idea have been developed with promising outcomes, such as the monoclonal antibody targeting interleukin-1β [45]. The anti-diabetic medications alleviate inflammation by differing degrees [46], which may also affect the diabetes-related myocardial remodeling and fibrosis, especially in the pressure-overloaded myocardium of AS patients. Additionally, whether individualizing the treatment strategy would be more beneficial in severe AS patients with concomitant diabetes should be evaluated in future trials.

This study has some limitations that needs to be considered. First, because of the shortage of resources, two separate imaging and biomarker cohorts were used for analysis, and analysis of the direct association of plasma proteins with noninvasive measures of fibrosis on CMR could not be performed. Second, information on the diabetes control status assessed by HbA1c or FBS values was not available for all patients and information on the duration of diabetes was unavailable. Thus, we used the diabetes medication status as a surrogate marker of the severity of diabetes, but its limitations must be acknowledged. Also, it should be considered that the number of patients on insulin was small to make any definite statements regarding the relationship between myocardial fibrosis and the type of medications for diabetes. Lastly, we used a select biomarker panel of 92 proteins, and future studies utilizing a more comprehensive set of proteins may provide more pathophysiological information as well as therapeutic targets.

## Conclusions

In conclusion, plasma proteome analyses indicate that diabetes is associated with increased systemic proinflammatory−profibrotic responses in patients with AS. These biological changes underlie the increase of myocardial fibrosis, advanced LV diastolic dysfunction, and ultimately, worse clinical outcomes observed in AS patients with concomitant diabetes.

## Supplementary Information

**Additional file 1. **Additional methods, additional figures S1–S5, additional table S1–S10.

## Data Availability

Individual-level participant data used in this study can be provided to researchers upon reasonable request to the corresponding author. A detailed proposal for how the data will be used is required.

## Abbreviations

AS: Aortic stenosis
AVR: Aortic valve replacement
CI: Confidence interval
CMR: Cardiac magnetic resonance
ECV: Extracellular volume fraction
HR: Hazard ratio
IHD: Ischemic heart disease
LGE: Late gadolinium enhancement
LV: Left ventricular
NPX: Normalized protein expression
OR: Odds ratio
