# Supplementary material for: Systemic proinflammatory−profibrotic response in aortic stenosis patients with diabetes and its relationship with myocardial remodeling and clinical outcome
Source: Cardiovasc Diabetol. 2023 Feb 10;22:30. doi: 10.1186/s12933-023-01763-1 (PMC9921197; doi:10.1186/s12933-023-01763-1)
Supplement: Supplementary file 1 — Additional file 1. Additional methods, additional figures S1–S5, additional table S1–S10. [file 12933_2023_1763_MOESM1_ESM.docx]

**Additional file 1**

**Systemic Proinflammatory−Profibrotic Response in Aortic Stenosis Patients with Diabetes and its Relationship with Myocardial Remodeling and Clinical Outcome**

Hyun-Jung Lee, MD, PhD^1^; Chan Soon Park, MD, PhD^1^; Sahmin Lee, MD, PhD^2^;
Jun-Bean Park, MD, PhD^1,3^; Hyung-Kwan Kim, MD, PhD^1,3^; Sung-Ji Park, MD, PhD^4^;
Yong-Jin Kim, MD, PhD^1,3^; Seung-Pyo Lee, MD, PhD^1,3,5^

^1^Division of Cardiology, Department of Internal Medicine, Seoul National University Hospital, Seoul, South Korea; ^2^Division of Cardiology, Department of Internal Medicine, Asan Medical Center, University of Ulsan College of Medicine, Seoul, South Korea; ^3^Department of Internal Medicine, Seoul National University College of Medicine, Seoul, South Korea; ^4^Division of Cardiology, Department of Medicine, Cardiovascular Imaging Center, Heart Vascular Stroke Institute, Samsung Medical Center, Sungkyunkwan University School of Medicine, Seoul, South Korea; ^5^Center for Precision Medicine, Seoul National University Hospital, Seoul, South Korea.

**Table of Contents**

1. Additional Methods
2. Additional Figures S1–5
3. Additional Tables S1–10

**ADDITIONAL METHODS**

***Study enrollment criteria and follow-up for the imaging cohort***

Consecutive patients with moderate or severe aortic stenosis (AS) were enrolled prospectively from three large-volume tertiary medical centers in Korea (Seoul National University Hospital [n=146], Asan Medical Center [n=40], and Samsung Medical Center [n=66]). All patients underwent cardiac magnetic resonance (CMR) imaging with T1 mapping performed both before and following intravenous gadolinium contrast administration.

In Seoul National University Hospital, patients with moderate or severe AS were enrolled prospectively from October 2011 to November 2015 (n=126), and from April 2019 to August 2020 (n=20). The enrollment criteria were moderate or severe AS defined by echocardiography as transaortic peak velocity ≥3.0 m/s or transaortic mean pressure gradient ≥30 mmHg, and aortic valve area of <1.5 cm^2^. For patients with left ventricular ejection fraction <40%, only the criteria of aortic valve area of <1.5 cm^2^ used. The exclusion criteria were concomitant valvular disease of at least moderate severity other than AS or non-cardiac comorbid conditions of life expectancy <1 year, serum creatinine >2.0 mg/dL or calculated creatinine clearance <30 ml/min/1.73m^2^, presence of artificial cochlear or permanent pacemaker, previous history of significant side effects after magnetic resonance imaging, chronic treatment with oral, intravenous, or intra-articular corticosteroids, untreated hyperthyroidism or hypothyroidism with thyroid-stimulating hormone levels more than 2 times upper limit of normal, women who are pregnant or breast-feeding, and history of chronic obstructive pulmonary disease or asthma on bronchodilators including long-acting beta2-agonist, anticholinergics, or inhaled steroids or recent acute aggravation of chronic obstructive pulmonary disease in the past 6 months. Last clinical follow-up or death was checked on August 13^th^, 2021.

In Asan Medical Center, patients with severe AS according to the guidelines awaiting aortic valve replacement were enrolled from June 2012 to January 2016 (n=40). Exclusion criteria were the presence of an implantable cardiac device, advanced renal dysfunction (estimated glomerular ﬁltration rate <30 ml/min/1.73m^2^), previous valve replacement, and presence of another coexistent myocardial pathology such as cardiac amyloidosis, hypertrophic cardiomyopathy, or myocarditis. Last clinical follow-up or death was checked on January 18^th^, 2021.

In Samsung Medical Center, patients with severe AS with preserved systolic function awaiting aortic valve replacement were enrolled from June 2012 to March 2015 (n=66). The enrollment criteria were severe AS with preserved systolic function deﬁned as indexed aortic valve area (AVA) <0.6 cm^2^/m^2^ and left ventricular ejection fraction ≥50%. The exclusion criteria were concomitant valvular disease of at least moderate severity other than AS, previous aortic valve replacement, obstructive epicardial coronary artery disease (>30% luminal stenosis in at least 1 coronary artery on coronary angiography), history of myocardial infarction or acute coronary syndrome; any absolute contraindication to CMR, or estimated glomerular ﬁltration rate <30 ml/min/1.73m^2^. Last clinical follow-up or death was checked on March 1^st^, 2020.

***Study enrollment criteria and sample collection for the biomarker cohort***

In Seoul National University Hospital, patients with severe AS undergoing aortic valve replacement were enrolled prospectively from March 2018 to June 2021 (n=100). After informed consent, 10 cc of patient blood was collected in EDTA-coated tubes and separated into plasma and buffy coat layers by centrifugation. The plasma and buffy coat samples were stored in EDTA-coated tubes in a deep freezer at -80℃, and the plasma samples were used for this study. The enrollment criteria were degenerative or bicuspid AS diagnosed on echocardiography, and exclusion criteria were AS due to rheumatic valvular heart disease, endocarditis or congenital valvular heart disease other than bicuspid AV.

**ADDITIONAL FIGURES**

**
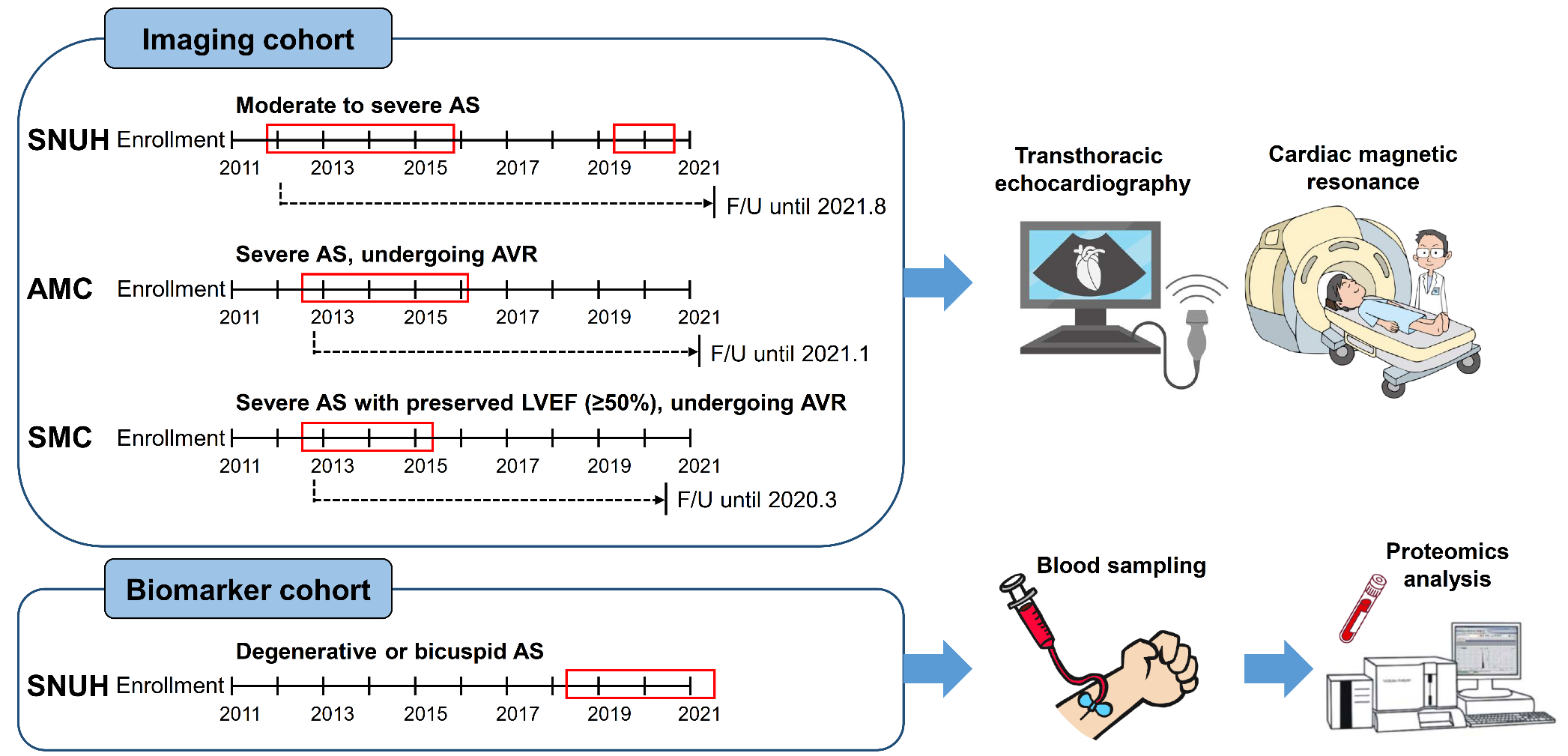
**

**Figure S1. Schematic diagram of study population.**

AMC, Asan Medical Center; AS, aortic stenosis; AVR, aortic valve replacement; F/U, follow-up; LVEF, left ventricular ejection fraction; SMC, Samsung Medical Center; SNUH, Seoul National University Hospital.

**Figure S2. Comparison of presence of LGE in AS patients according to diabetes and diabetes medication status.**

Comparison of the presence of late gadolinium enhancement (LGE) in (A) patients with versus without diabetes and in (B) patients stratified by the diabetes medication status.

AS, aortic stenosis; DM, diabetes mellitus; LGE, late gadolinium enhancement.

**
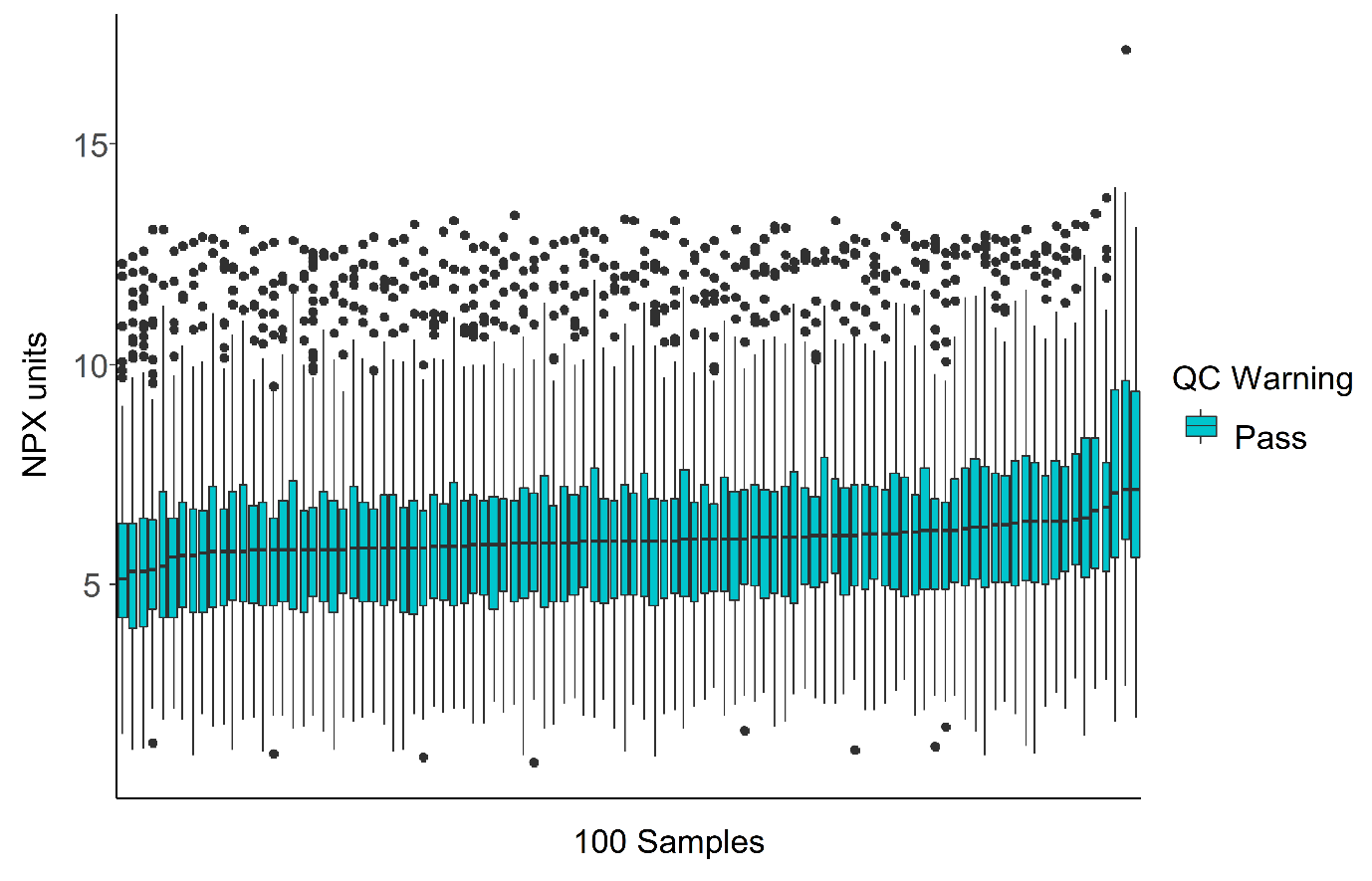
**

**Figure S3. Distribution of NPX values for each sample.**

All samples passed quality control and protein levels were normalized and presented in NPX (normalized protein expression) units using the Log_2_ scale (1 NPX difference equaling 2-fold change in protein concentration).

**Figure S4. Over-represented pathways in the plasma proteome of AS patients with diabetes: exploratory analysis with alternative criteria.**

(A) Fifteen biomarkers fulfilled the criteria of nominal p-value <0.050 and minimal fold change of 1.15. (B) Pathway over-representation analyses with these plasma biomarkers were perfomed using g:Profiler with Gene Ontology terms. The sizes of circles signify intersection size. Refer to Table S8 for the list of enriched pathways.

adj, adjusted; AS, aortic stenosis; BP, biological process; CC, cellular component; GO, Gene Ontology domains; MF, molecular function.

**
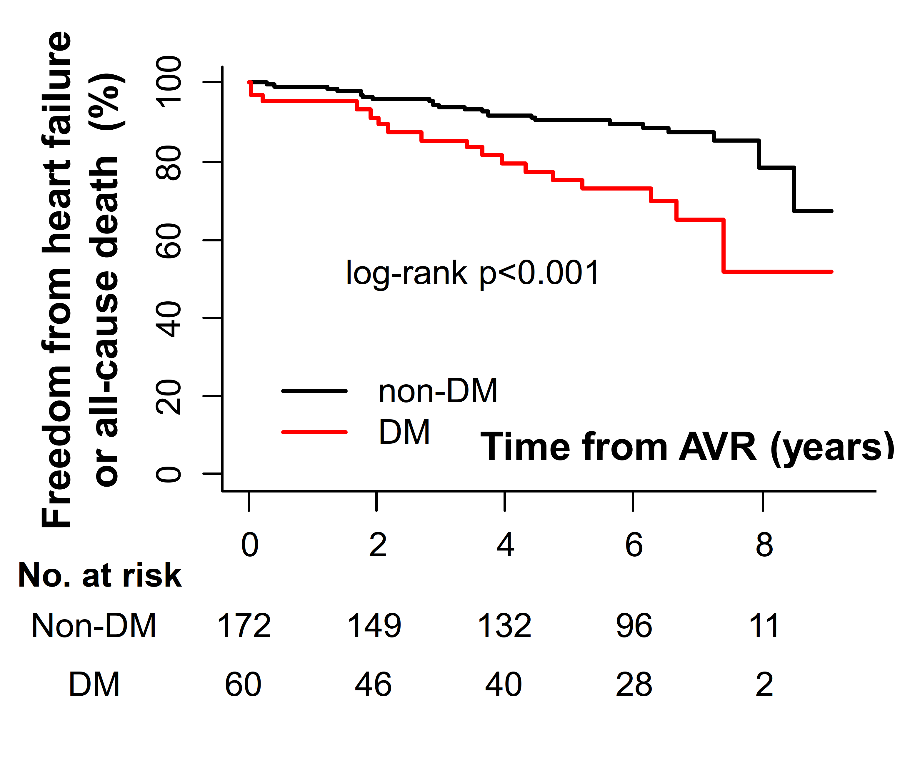
**

**Figure S5. Comparison of event-free survival in AS patients according to diabetes, after AVR.**

Kaplan-Meier analysis with p-values by the log-rank test are presented for the composite outcome of admission for heart failure and all-cause death.

AS, aortic stenosis; AVR, aortic valve replacement; DM, diabetes mellitus.

**ADDITIONAL TABLES**

**Table S1. Technical details of cardiovascular magnetic resonance by study centers.**

| Site | Scanner | Pulse sequence (pre) | Pulse sequence (post) | Contrast agent, dose and timing | N | Mean native T1, ms | Mean ECV% | Mean LGE% |
| --- | --- | --- | --- | --- | --- | --- | --- | --- |
| Seoul National University Hospital, Seoul, Korea | Siemens Trio 3T | MOLLI  3(3)-3(3)-5 | MOLLI  3(3)-3(3)-5 | Magnevist  0.20 mmol/kg  10 mins | 125 | 1232±53 | 27.9±3.3 | 1.0±2.8 |
| Seoul National University Hospital, Seoul, Korea | Siemens Skyra 3T | MOLLI  3(3)-3(3)-5 | MOLLI  3(3)-3(3)-5 | Dotarem  0.20 mmol/kg  15 mins | 21 | 1271±59 | 28.3±3.7 | 0.7±0.6 |
| Asan Medical Center, Seoul, Korea | Siemens Avanto 1.5T | MOLLI  3(3)-3(3)-5 | MOLLI  3(3)-3(3)-5 | Gadovist  0.10 mmol/kg  20 mins | 40 | 1000±39 | 26.3±2.3 | 0.5±1.7 |
| Samsung Medical Center, Seoul, Korea | Siemens Avanto 1.5T | MOLLI  5(3)-3 | MOLLI  4(1)-3(1)-2 | Gadobutrol  0.10 mmol/kg  15 mins | 66 | 992±60 | 26.3±2.4 | 1.5±3.1 |

ECV, extracellular volume fraction; LGE, late gadolinium enhancement; MOLLI, Modified Look-Locker inversion recovery.

**Table S2. List of protein biomarkers included in Cardiovascular Panel III v.6114 (Olink, Uppsala, Sweden).**

| **Target** | **Abbreviation** | **UniProt ID** |
| --- | --- | --- |
| Aminopeptidase N | AP-N | P15144 |
| Azurocidin | AZU1 | P20160 |
| Bleomycin hydrolase | BLM hydrolase | Q13867 |
| Cadherin-5 | CDH5 | P33151 |
| Carboxypeptidase A1 | CPA1 | P15085 |
| Carboxypeptidase B | CPB1 | P15086 |
| Caspase-3 | CASP-3 | P42574 |
| Cathepsin D | CTSD | P07339 |
| Cathepsin Z | CTSZ | Q9UBR2 |
| C-C motif chemokine 15 | CCL15 | Q16663 |
| C-C motif chemokine 16 | CCL16 | O15467 |
| C-C motif chemokine 24 | CCL24 | O00175 |
| CD166 antigen | ALCAM | Q13740 |
| Chitinase-3-like protein 1 | CHI3L1 | P36222 |
| Chitotriosidase-1 | CHIT1 | Q13231 |
| Collagen alpha-1(I) chain | COL1A1 | P02452 |
| Complement component C1q receptor | CD93 | Q9NPY3 |
| Contactin-1 | CNTN1 | Q12860 |
| C-X-C motif chemokine 16 | CXCL16 | Q9H2A7 |
| Cystatin-B | CSTB | P04080 |
| Elafin | PI3 | P19957 |
| Ephrin type-B receptor 4 | EPHB4 | P54760 |
| Epidermal growth factor receptor | EGFR | P00533 |
| Epithelial cell adhesion molecule | Ep-CAM | P16422 |
| E-selectin | SELE | P16581 |
| Fatty acid-binding protein, adipocyte | FABP4 | P15090 |
| Galectin-3 | Gal-3 | P17931 |
| Galectin-4 | Gal-4 | P56470 |
| Granulins | GRN | P28799 |
| Growth/differentiation factor 15 | GDF-15 | Q99988 |
| Insulin-like growth factor-binding protein 1 | IGFBP-1 | P08833 |
| Insulin-like growth factor-binding protein 2 | IGFBP-2 | P18065 |
| Insulin-like growth factor-binding protein 7 | IGFBP-7 | Q16270 |
| Integrin beta-2 | ITGB2 | P05107 |
| Intercellular adhesion molecule | ICAM-2 | P13598 |
| Interleukin-1 receptor type 1 | IL-1RT1 | P14778 |
| Interleukin-1 receptor type 2 | IL-1RT2 | P27930 |
| Interleukin-17 receptor A | IL-17RA | Q96F46 |
| Interleukin-18-binding protein | IL-18BP | O95998 |
| Interleukin-2 receptor subunit alpha | IL2-RA | P01589 |
| Interleukin-6 receptor subunit alpha | IL-6RA | P08887 |
| Junctional adhesion molecule A | JAM-A | Q9Y624 |
| Kallikrein-6 | KLK6 | Q92876 |
| Low-density lipoprotein receptor | LDL receptor | P01130 |
| Lymphotoxin-beta receptor | LTBR | P36941 |
| Matrix extracellular phosphoglycoprotein | MEPE | Q9NQ76 |
| Matrix metalloproteinase-2 | MMP-2 | P08253 |
| Matrix metalloproteinase-3 | MMP-3 | P08254 |
| Matrix metalloproteinase-9 | MMP-9 | P14780 |
| Metalloproteinase inhibitor 4 | TIMP4 | Q99727 |
| Monocyte chemotactic protein 1 | MCP-1 | P13500 |
| Myeloblastin | PRTN3 | P24158 |
| Myeloperoxidase | MPO | P05164 |
| Myoglobin | MB | P02144 |
| Neurogenic locus notch homolog protein 3 | Notch 3 | Q9UM47 |
| N-terminal prohormone brain natriuretic peptide | NT-proBNP | NA |
| Osteopontin | OPN | P10451 |
| Osteoprotegerin | OPG | O00300 |
| Paraoxonase | PON3 | Q15166 |
| Peptidoglycan recognition protein 1 | PGLYRP1 | O75594 |
| Perlecan | PLC | P98160 |
| Plasminogen activator inhibitor 1 | PAI | P05121 |
| Platelet endothelial cell adhesion molecule | PECAM-1 | P16284 |
| Platelet glycoprotein VI | GP6 | Q9HCN6 |
| Platelet-derived growth factor subunit A | PDGF subunit A | P04085 |
| Proprotein convertase subtilisin/kexin type 9 | PCSK9 | Q8NBP7 |
| Protein delta homolog 1 | DLK-1 | P80370 |
| P-selectin | SELP | P16109 |
| Pulmonary surfactant-associated protein D | PSP-D | P35247 |
| Resistin | RETN | Q9HD89 |
| Retinoic acid receptor responder protein 2 | RARRES2 | Q99969 |
| Scavenger receptor cysteine-rich type protein M130 | CD163 | Q86VB7 |
| Secretoglobin family 3A member 2 | SCGB3A2 | Q96PL1 |
| Spondin-1 | SPON1 | Q9HCB6 |
| ST2 protein | ST2 | Q01638 |
| Tartrate-resistant acid phosphatase type 5 | TR-AP | P13686 |
| Tissue factor pathway inhibitor | TFPI | P10646 |
| Tissue-type plasminogen activator | t-PA | P00750 |
| Transferrin receptor protein 1 | TR | P02786 |
| Trefoil factor 3 | TFF3 | Q07654 |
| Trem-like transcript 2 protein | TLT-2 | Q5T2D2 |
| Tumor necrosis factor ligand superfamily member 13B | TNFSF13B | Q9Y275 |
| Tumor necrosis factor receptor 1 | TNF-R1 | P19438 |
| Tumor necrosis factor receptor 2 | TNF-R2 | P20333 |
| Tumor necrosis factor receptor superfamily member 10C | TNFRSF10C | O14798 |
| Tumor necrosis factor receptor superfamily member 14 | TNFRSF14 | Q92956 |
| Tumor necrosis factor receptor superfamily member 6 | FAS | P25445 |
| Tyrosine-protein kinase receptor UFO | AXL | P30530 |
| Tyrosine-protein phosphatase non-receptor type substrate 1 | SHPS-1 | P78324 |
| Urokinase plasminogen activator surface receptor | U-PAR | Q03405 |
| Urokinase-type plasminogen activator | uPA | P00749 |
| von Willebrand factor | vWF | P04275 |

**Table S3. Comparison of patients in the imaging cohort and biomarker cohort.**

|  | **Imaging cohort (n=253)** | **Biomarker cohort (n=100)** | **p-value** |
| --- | --- | --- | --- |
| Diabetes mellitus | 66 (26.1) | 27 (27.0) | 0.967 |
| Age (years) | 67.7 ± 9.5 | 66.6 ± 9.6 | 0.335 |
| Male | 127 (50.2) | 60 (60.0) | 0.123 |
| Hypertension | 151 (59.7) | 58 (58.0) | 0.865 |
| Atrial fibrillation | 31 (12.3) | 13 (13.0) | 0.990 |
| Stroke | 21 (8.3) | 11 (11.0) | 0.555 |
| Ischemic heart disease | 56 (22.1) | 19 (19.0) | 0.614 |
| Creatinine (mg/dL) | 0.88 ± 0.23 | 1.12 ± 1.37 | 0.082 |
| Euroscore II | 1.6 ± 1.5 | 1.7 ± 1.8 | 0.590 |
| NYHA III-IV | 55 (21.8) | 16 (16.0) | 0.280 |
| Medication |  |  |  |
| ACE inhibitor/ARB | 111 (43.9) | 37 (37.0) | 0.289 |
| Beta-blocker | 116 (45.8) | 48 (48.0) | 0.805 |
| Echocardiography |  |  |  |
| LV end-diastolic dimension (mm) | 50.2 ± 6.7 | 48.8 ± 6.9 | 0.069 |
| LV end-systolic dimension (mm) | 31.8 ± 7.8 | 31.3 ± 7.0 | 0.626 |
| LV mass index (g/m^2^) | 132 ± 40 | 128 ± 55 | 0.570 |
| Relative wall thickness | 0.44 ± 0.09 | 0.46 ± 0.09 | 0.084 |
| LV ejection fraction (%) | 59.6 ± 9.9 | 59.9 ± 9.2 | 0.813 |
| Left atrial diameter (mm) | 43.8 ± 6.9 | 43.8 ± 8.5 | 0.996 |
| E velocity (m/s) | 0.79 ± 0.38 | 0.74 ± 0.35 | 0.252 |
| A velocity (m/s) | 0.87 ± 0.29 | 0.85 ± 0.25 | 0.612 |
| Deceleration time (ms) | 247 ± 79 | 243 ± 96 | 0.737 |
| E/A | 0.78 [0.63-1.10] | 0.75 [0.59-0.90] | 0.243 |
| e′ velocity (cm/s) | 4.6 ± 1.4 | 4.6 ± 1.5 | 0.771 |
| a′ velocity (cm/s) | 7.3 ± 1.8 | 7.1 ± 1.9 | 0.529 |
| s′ velocity (cm/s) | 5.1 ± 1.4 | 4.9 ± 1.4 | 0.220 |
| E/e′ | 15.7 [12.3-21.2] | 14.3 [11.3-20.8] | 0.123 |
| TR Vmax (m/s) | 2.5 ± 0.4 | 2.5 ± 0.5 | 0.996 |
| PASP (mmHg) | 34.4 ± 9.4 | 36.2 ± 11.2 | 0.194 |
| Left atrial volume index (mL/m^2^) | 49.2 [39.0-62.7] | 41.5 [32.9-51.1] | <0.001 |
| Peak aortic velocity (m/s) | 4.7 ± 0.8 | 4.6 ± 0.8 | 0.140 |
| AV mean PG (mmHg) | 55 ± 21 | 53 ± 18 | 0.335 |
| AV area (cm^2^) | 0.76 ± 0.23 | 0.78 ± 0.26 | 0.433 |
| Presence of LVDD (n=318) | 139 (60.2) | 41 (47.1) | 0.049 |

ACE, angiotensin converting enzyme; ARB, angiotensin receptor blocker; AV, aortic valve; DM, diabetes mellitus; LV, left ventricular; LVDD, LV diastolic dysfunction; NYHA, New York Heart Association; PASP, pulmonary artery systolic pressure; PG, pressure gradient; Vmax, maximal velocity.

**Table S4. Comparison of patients with diabetes in the imaging cohort and biomarker cohort.**

|  | **DM patients in  imaging cohort (N=66)** | **DM patients in  biomarker cohort (N=27)** | **p-value** |
| --- | --- | --- | --- |
| Age (years) | 70.4 ± 6.8 | 69.5 ± 8.2 | 0.591 |
| Male | 34 (51.5) | 17 (63.0) | 0.437 |
| Hypertension | 48 (72.7) | 19 (70.4) | >0.999 |
| Atrial fibrillation | 13 (19.7) | 2 (7.4) | 0.249 |
| Stroke | 8 (12.1) | 5 (18.5) | 0.633 |
| Ischemic heart disease | 25 (37.9) | 9 (33.3) | 0.860 |
| Creatinine (mg/dL) | 0.91 ± 0.30 | 1.25 ± 1.80 | 0.342 |
| Euroscore II | 2.5 ± 2.5 | 1.8 ± 2.3 | 0.235 |
| NYHA III-IV | 19 (28.8) | 3 (11.1) | 0.121 |
| Medication |  |  |  |
| ACE inhibitor/ARB | 36 (54.5) | 12 (44.4) | 0.512 |
| Beta-blocker | 28 (42.4) | 15 (55.6) | 0.356 |
| Diabetes medication |  |  | 0.658 |
| None | 13 (19.7) | 4 (14.8) |  |
| Oral medication only | 47 (71.2) | 19 (70.4) |  |
| Insulin user | 6 (9.1) | 4 (14.8) |  |
| HbA1c | 6.6 [6.2-7.5] (n=58) | 7.0 [6.4-7.4] | 0.205 |
| FBS | 132 [110-172] (n=52) | 120 [107-147] (n=25) | 0.215 |
| Echocardiography |  |  |  |
| LV end-diastolic dimension (mm) | 50.6 ± 6.4 | 48.3 ± 6.0 | 0.115 |
| LV end-systolic dimension (mm) | 32.4 ± 9.0 | 31.2 ± 7.4 | 0.536 |
| LV mass index (g/m^2^) | 130 ± 37 | 124 ± 42 | 0.485 |
| Relative wall thickness | 0.44 ± 0.09 | 0.47 ± 0.07 | 0.211 |
| LV ejection fraction (%) | 57.6 ± 12.0 | 57.9 ± 9.5 | 0.925 |
| Left atrial diameter (mm) | 44.8 ± 6.2 | 44.6 ± 7.4 | 0.926 |
| E velocity (m/s) | 0.88 ± 0.43 | 0.65 ± 0.27 | 0.004 |
| A velocity (m/s) | 0.90 ± 0.26 | 0.93 ± 0.18 | 0.574 |
| Deceleration time (ms) | 229 ± 64 | 230 ± 80 | 0.931 |
| E/A | 0.78 [0.60-1.10] | 0.65 [0.56-0.79] | 0.031 |
| e′ velocity (cm/s) | 4.2 ± 1.4 | 4.0 ± 1.1 | 0.478 |
| a′ velocity (cm/s) | 7.2 ± 2.1 | 7.2 ± 1.8 | 0.982 |
| s′ velocity (cm/s) | 4.9 ± 1.5 | 4.8 ± 1.4 | 0.879 |
| E/e′ | 19.2 [13.3-31.0] | 16.6 [13.8-20.0] | 0.111 |
| TR Vmax (m/s) | 2.7 ± 0.6 | 2.5 ± 0.5 | 0.209 |
| PASP (mmHg) | 38.0 ± 12.4 | 35.0 ± 10.6 | 0.446 |
| Left atrial volume index (mL/m^2^) | 48.0 [41.2-58.0] | 38.4 [32.6-47.6] | 0.006 |
| Peak aortic velocity (m/s) | 4.5 ± 0.9 | 4.6 ± 0.7 | 0.732 |
| AV mean PG (mmHg) | 51 ± 21 | 54 ± 17 | 0.546 |
| AV area (cm^2^) | 0.74 ± 0.25 | 0.74 ± 0.20 | 0.937 |
| Presence of LVDD (n=84) | 47 (79.7) | 15 (60.0) | 0.109 |

ACE, angiotensin converting enzyme; ARB, angiotensin receptor blocker; AV, aortic valve; DM, diabetes mellitus; FBS, fasting blood sugar; LV, left ventricular; LVDD, LV diastolic dysfunction; NYHA, New York Heart Association; PASP, pulmonary artery systolic pressure; PG, pressure gradient; Vmax, maximal velocity.

**Table S5. Baseline diabetes medication of the patients with diabetes in the study population.**

|  | **Insulin** | **Metformin** | **Sulfonylurea** | **DPP4i** | **SGLT2i** | **AGi** | **TZD** |
| --- | --- | --- | --- | --- | --- | --- | --- |
| **Imaging cohort**  **(n=66)** | 6 (9.1) | 34 (51.5) | 25 (37.9) | 23 (34.8) | 0 (0) | 2 (3.0) | 2 (3.0) |
| **Biomarker cohort**  **(n=27)** | 4 (14.8) | 17 (63.0) | 6 (22.2) | 14 (51.9) | 3 (11.1) | 0 (0) | 0 (0) |

DPP4i, dipeptidyl peptidase-4 inhibitors; SGLT2i, sodium-glucose cotransporter-2 inhibitors; AGi, alpha-glucosidase inhibitors; TZD, thiazolidinediones

**Table S6. White blood cell count in patients with and without diabetes in the biomarker cohort.**

|  | **Total (n=100)** | **Non-DM (N=73)** | **DM (N=27)** | **p-value** |
| --- | --- | --- | --- | --- |
| White blood cell count | 9665 ± 3536 | 9821 ± 3631 | 9231 ± 3286 | 0.469 |
| Neutrophil count | 7800 ± 3351 | 7945 ± 3431 | 7409 ± 3159 | 0.489 |
| Lymphocyte count | 1247 ± 681 | 1249 ± 719 | 1242 ± 579 | 0.964 |
| Neutrophil/lymphocyte ratio | 8.2 ± 5.4 | 8.3 ± 5.0 | 8.1 ± 6.3 | 0.867 |

DM, diabetes mellitus

**Table S7. Echocardiography analysis of the patients with and without diabetes in the biomarker cohort.**

|  | **Total (n=100)** | **Non-DM (N=73)** | **DM (N=27)** | **p-value** |
| --- | --- | --- | --- | --- |
| Echocardiography |  |  |  |  |
| LV end-diastolic dimension (mm) | 48.8 ± 6.9 | 49.0 ± 7.3 | 48.3 ± 6.0 | 0.666 |
| LV end-systolic dimension (mm) | 31.3 ± 7.0 | 31.4 ± 6.9 | 31.2 ± 7.4 | 0.907 |
| LV mass index (g/m^2^) | 128 ± 55 | 130 ± 59 | 124 ± 42 | 0.599 |
| Relative wall thickness | 0.46 ± 0.09 | 0.46 ± 0.09 | 0.47 ± 0.07 | 0.683 |
| LV ejection fraction (%) | 59.9 ± 9.2 | 60.6 ± 9.1 | 57.9 ± 9.5 | 0.203 |
| Left atrial diameter (mm) | 43.8 ± 8.5 | 43.5 ± 8.9 | 44.6 ± 7.4 | 0.561 |
| E velocity (m/s) | 0.74 ± 0.35 | 0.77 ± 0.37 | 0.65 ± 0.27 | 0.168 |
| A velocity (m/s) | 0.85 ± 0.25 | 0.82 ± 0.26 | 0.93 ± 0.18 | 0.054 |
| Deceleration time (ms) | 243 ± 96 | 248 ± 102 | 230 ± 80 | 0.431 |
| E/A | 0.75 [0.59-0.90] | 0.83 [0.62-1.00] | 0.65 [0.56-0.79] | 0.029 |
| e′ velocity (cm/s) | 4.6 ± 1.5 | 4.9 ± 1.6 | 4.0 ± 1.1 | 0.016 |
| a′ velocity (cm/s) | 7.1 ± 1.9 | 7.1 ± 1.9 | 7.2 ± 1.8 | 0.959 |
| s′ velocity (cm/s) | 4.9 ± 1.4 | 4.9 ± 1.4 | 4.8 ± 1.4 | 0.911 |
| E/e′ | 14.3 [11.3-20.8] | 13.3 [10.9-22.0] | 16.6 [13.8-20.0] | 0.198 |
| TR Vmax (m/s) | 2.5 ± 0.5 | 2.5 ± 0.5 | 2.5 ± 0.5 | 0.668 |
| PASP (mmHg) | 36.2 ± 11.2 | 36.6 ± 11.5 | 35.0 ± 10.6 | 0.673 |
| Left atrial volume index (mL/m^2^) | 41.5 [32.9-51.1] | 41.7 [33.0-52.8] | 38.4 [32.6-47.6] | 0.616 |
| Peak aortic velocity (m/s) | 4.6 ± 0.8 | 4.6 ± 0.8 | 4.6 ± 0.7 | 0.781 |
| AV mean PG (mmHg) | 53 ± 18 | 52 ± 18 | 54 ± 17 | 0.992 |
| AV area (cm^2^) | 0.78 ± 0.26 | 0.79 ± 0.27 | 0.74 ± 0.20 | 0.826 |
| AS severity (by AV area) |  |  |  | 0.307 |
| Severe AS | 86 (87.8) | 61 (84.7) | 25 (96.2) |  |
| Moderate AS | 11 (11.2) | 10 (13.9) | 1 (3.8) |  |
| Mild AS | 1 (1.0) | 1 (1.4) | 0 (0) |  |
| Presence of LVDD (n=87) | 41 (47.1) | 26 (41.9) | 15 (60.0) | 0.197 |
| LVDD grade (n=77) |  |  |  | 0.309 |
| Normal | 28 (36.4) | 22 (40.0) | 6 (27.3) |  |
| Indeterminate | 18 (23.4) | 14 (25.5) | 4 (18.2) |  |
| Grade 1 LVDD | 4 (5.2) | 3 (5.5) | 1 (4.5) |  |
| Grade 2 LVDD | 25 (32.5) | 14 (25.5) | 11 (50.0) |  |
| Grade 3 LVDD | 2 (2.6) | 2 (3.6) | 0 (0) |  |

AV, aortic valve; LV, left ventricular; LVDD, LV diastolic dysfunction; PASP, pulmonary artery systolic pressure; PG, pressure gradient; Vmax, maximal velocity.

**Table S8. Comparison of myocardial fibrosis and left ventricular diastolic function according to diabetes medication in aortic stenosis patients.**

|  | **Non-DM (n=187)** | **DM (no medication) (n=13)** | **DM (OHA only) (n=47)** | **DM (insulin) (n=6)** | **p-value** | **p-for-trend** |
| --- | --- | --- | --- | --- | --- | --- |
| Presence of LVDD | 92 (53.5) | 8 (80.0) | 33 (76.7) | 6 (100.0) | 0.003 | <0.001 |
| Presence of LGE | 75 (40.1) | 7 (53.8) | 25 (53.2) | 5 (83.3) | 0.071 | 0.016 |
| LGE (%) | 0.0 [0.0-0.5] | 0.3 [0.0-0.8] | 0.3 [0.0-1.6] | 0.8 [0.0-1.5] | 0.063 | 0.070 |
| ECV (%) | 26.7 [24.9-28.5] | 26.1 [25.1-27.9] | 27.8 [25.6-29.6] | 30.2 [29.8-31.1] | 0.005 | 0.003 |

DM, diabetes mellitus; ECV, extracellular volume fraction; LGE, late gadolinium enhancement; LVDD, left ventricular diastolic dysfunction; OHA, oral hypoglycemic agents.

**Table S9. Comparison of myocardial fibrosis and left ventricular diastolic function according to HbA1c levels in the subset of aortic stenosis patients with measurements (n=162).**

|  | **HbA1c <6.0% (n=89)** | **HbA1c ≥6.0 and <6.5% (n=37)** | **HbA1c ≥6.5% (n=36)** | **p-value** | **p-for-trend** |
| --- | --- | --- | --- | --- | --- |
| Presence of LVDD | 41 (48.2) | 22 (64.7) | 29 (82.9) | 0.002 | <0.001 |
| Presence of LGE | 40 (44.9) | 19 (51.4) | 20 (55.6) | 0.526 | 0.260 |
| LGE (%) | 0.1 [0.0-0.7] | 0.3 [0.0-1.2] | 0.4 [0.0-2.0] | 0.165 | 0.114 |
| ECV (%) | 26.2 [24.9-28.5] | 27.0 [25.3-28.3] | 27.9 [25.9-29.6] | 0.102 | 0.073 |

DM, diabetes mellitus; ECV, extracellular volume fraction; LGE, late gadolinium enhancement; LVDD, left ventricular diastolic dysfunction.

**Table S10. Comparison of myocardial fibrosis and left ventricular diastolic function according to fasting blood glucose levels in the subset of aortic stenosis patients with measurements (n=137).**

|  | **FBS <100 mg/dL (n=39)** | **FBS ≥100 and <125 mg/dL (n=43)** | **FBS ≥125 mg/dL (n=55)** | **p-value** | **p-for-trend** |
| --- | --- | --- | --- | --- | --- |
| Presence of LVDD | 20 (55.6) | 24 (64.9) | 36 (73.5) | 0.227 | 0.085 |
| Presence of LGE | 14 (35.9) | 22 (51.2) | 34 (61.8) | 0.047 | 0.014 |
| LGE (%) | 0.1 [0.0-0.7] | 0.1 [0.0-0.4] | 0.4 [0.0-1.5] | 0.067 | 0.872 |
| ECV (%) | 25.8 [24.5-27.6] | 26.2 [24.2-28.4] | 27.0 [25.5-28.8] | 0.083 | 0.047 |

DM, diabetes mellitus; ECV, extracellular volume fraction; LGE, late gadolinium enhancement; LVDD, left ventricular diastolic dysfunction.

**Table S11. Comparison of plasma biomarker expression in aortic stenosis patients with and without diabetes**

| **Biomarker** | **DM^a^ (n=27)** | **Non-DM^a^ (n=73)** | **Difference (log_2_ scale)^a^** | **Nominal p-value** | **Adjusted p-value^b^** |
| --- | --- | --- | --- | --- | --- |
| SELE | 12.069 | 11.552 | 0.518 | 0.000125 | 0.0115 |
| IL-1RT1 | 6.647 | 6.359 | 0.289 | 0.000307 | 0.0141 |
| CTSD | 4.03 | 3.721 | 0.309 | 0.001695 | 0.0468 |
| Gal-4 | 5.805 | 5.436 | 0.369 | 0.002258 | 0.0468 |
| Gal-3 | 5.995 | 5.745 | 0.25 | 0.002692 | 0.0468 |
| IL-1RT2 | 6.931 | 6.629 | 0.303 | 0.003254 | 0.0468 |
| GDF-15 | 6.648 | 6.132 | 0.516 | 0.004526 | 0.0468 |
| ICAM-2 | 5.883 | 5.625 | 0.257 | 0.004532 | 0.0468 |
| ITGB2 | 6.604 | 6.321 | 0.283 | 0.004578 | 0.0468 |
| IL-18BP | 7.095 | 6.825 | 0.27 | 0.00694 | 0.0638 |
| CCL16 | 8.005 | 7.607 | 0.398 | 0.007904 | 0.0661 |
| ALCAM | 7.77 | 7.627 | 0.143 | 0.017705 | 0.1357 |
| t-PA | 6.04 | 5.475 | 0.565 | 0.026639 | 0.1885 |
| CD163 | 9.268 | 9.067 | 0.201 | 0.031407 | 0.2064 |
| PSP-D | 3.795 | 3.323 | 0.472 | 0.034512 | 0.2117 |
| IGFBP-1 | 7.278 | 6.703 | 0.575 | 0.048772 | 0.2804 |
| TR-AP | 5.964 | 5.783 | 0.181 | 0.064941 | 0.3514 |
| GRN | 6.098 | 5.953 | 0.145 | 0.072296 | 0.3695 |
| U-PAR | 6.394 | 6.119 | 0.275 | 0.079020 | 0.3826 |
| AP-N | 6.508 | 6.288 | 0.22 | 0.104007 | 0.4411 |
| COL1A1 | 2.632 | 2.811 | -0.179 | 0.106867 | 0.4411 |
| TFF3 | 5.687 | 5.36 | 0.327 | 0.122482 | 0.4411 |
| CHIT1 | 5.106 | 4.41 | 0.696 | 0.126369 | 0.4411 |
| IL2-RA | 3.859 | 3.684 | 0.175 | 0.131932 | 0.4411 |
| uPA | 6.079 | 5.905 | 0.174 | 0.133337 | 0.4411 |
| TNF-R2 | 6.724 | 6.475 | 0.248 | 0.135364 | 0.4411 |
| PON3 | 6.536 | 6.721 | -0.185 | 0.139563 | 0.4411 |
| AXL | 9.72 | 9.587 | 0.133 | 0.141181 | 0.4411 |
| EPHB4 | 6.55 | 6.387 | 0.163 | 0.144614 | 0.4411 |
| FABP4 | 6.011 | 5.63 | 0.382 | 0.149363 | 0.4411 |
| MCP-1 | 5.115 | 4.948 | 0.167 | 0.154609 | 0.4411 |
| IL-6RA | 12.831 | 12.728 | 0.103 | 0.159303 | 0.4411 |
| TLT-2 | 6.165 | 6.014 | 0.15 | 0.164687 | 0.4411 |
| CTSZ | 5.115 | 4.991 | 0.123 | 0.166368 | 0.4411 |
| CPA1 | 6.46 | 6.243 | 0.217 | 0.167803 | 0.4411 |
| SELP | 9.969 | 9.79 | 0.178 | 0.194034 | 0.4665 |
| TNFRSF10C | 6.378 | 6.255 | 0.123 | 0.194516 | 0.4665 |
| PI3 | 3.312 | 3.038 | 0.275 | 0.196813 | 0.4665 |
| FAS | 7.053 | 6.848 | 0.206 | 0.197742 | 0.4665 |
| TNFRSF14 | 5.028 | 4.779 | 0.249 | 0.204557 | 0.4705 |
| CPB1 | 6.579 | 6.403 | 0.176 | 0.247469 | 0.5553 |
| OPG | 4.411 | 4.286 | 0.124 | 0.255617 | 0.5599 |
| SHPS-1 | 4.255 | 4.139 | 0.116 | 0.271995 | 0.5638 |
| PECAM-1 | 5.306 | 5.169 | 0.137 | 0.278700 | 0.5638 |
| TNFSF13B | 7.763 | 7.657 | 0.105 | 0.281006 | 0.5638 |
| CSTB | 4.881 | 4.635 | 0.245 | 0.281898 | 0.5638 |
| CDH5 | 4.997 | 4.901 | 0.096 | 0.290560 | 0.5688 |
| SCGB3A2 | 3.358 | 3.129 | 0.229 | 0.300397 | 0.5719 |
| PAI | 6.358 | 6.09 | 0.268 | 0.308872 | 0.5719 |
| Ep-CAM | 5.812 | 5.984 | -0.171 | 0.314721 | 0.5719 |
| RETN | 7.058 | 6.852 | 0.206 | 0.320845 | 0.5719 |
| TNF-R1 | 6.965 | 6.767 | 0.199 | 0.326699 | 0.5719 |
| GP6 | 2.803 | 2.672 | 0.131 | 0.329485 | 0.5719 |
| Notch-3 | 6.188 | 6.102 | 0.086 | 0.339526 | 0.5785 |
| CNTN1 | 4.364 | 4.282 | 0.082 | 0.361739 | 0.5963 |
| TR | 6.576 | 6.454 | 0.121 | 0.363591 | 0.5963 |
| PDGF-subunit-A | 3.012 | 2.832 | 0.179 | 0.369423 | 0.5963 |
| LTBR | 4.027 | 3.892 | 0.135 | 0.378894 | 0.6010 |
| CD93 | 10.558 | 10.479 | 0.079 | 0.418225 | 0.6455 |
| MEPE | 5.344 | 5.484 | -0.14 | 0.420988 | 0.6455 |
| RARRES2 | 12.194 | 12.133 | 0.06 | 0.439936 | 0.6635 |
| CXCL16 | 6.292 | 6.244 | 0.049 | 0.466297 | 0.6784 |
| JAM-A | 6.349 | 6.187 | 0.162 | 0.480881 | 0.6784 |
| IGFBP-7 | 11.335 | 11.265 | 0.07 | 0.483286 | 0.6784 |
| EGFR | 3.092 | 3.055 | 0.037 | 0.485156 | 0.6784 |
| TIMP4 | 4.392 | 4.308 | 0.084 | 0.486674 | 0.6784 |
| MMP-3 | 7.231 | 7.393 | -0.162 | 0.521093 | 0.7155 |
| IL-17RA | 5.027 | 4.983 | 0.045 | 0.536356 | 0.7257 |
| PRTN3 | 5.176 | 5.078 | 0.098 | 0.555518 | 0.7407 |
| PLC | 8.668 | 8.611 | 0.056 | 0.573384 | 0.7500 |
| MMP-9 | 5.842 | 5.748 | 0.094 | 0.578783 | 0.7500 |
| MMP-2 | 5.294 | 5.245 | 0.048 | 0.603947 | 0.7717 |
| MPO | 3.801 | 3.751 | 0.049 | 0.626645 | 0.7897 |
| ST2 | 5.66 | 5.561 | 0.099 | 0.661577 | 0.8121 |
| IGFBP-2 | 7.681 | 7.774 | -0.093 | 0.662013 | 0.8121 |
| PGLYRP1 | 8.229 | 8.171 | 0.058 | 0.675578 | 0.8178 |
| CCL24 | 6.313 | 6.363 | -0.05 | 0.692622 | 0.8275 |
| BLM-hydrolase | 2.81 | 2.748 | 0.062 | 0.711948 | 0.8397 |
| CCL15 | 8.514 | 8.441 | 0.073 | 0.726306 | 0.8458 |
| OPN | 9.221 | 9.265 | -0.044 | 0.771195 | 0.8766 |
| PCSK9 | 3.428 | 3.404 | 0.024 | 0.773831 | 0.8766 |
| NT-proBNP | 9.143 | 9.227 | -0.083 | 0.781331 | 0.8766 |
| KLK6 | 4.659 | 4.631 | 0.028 | 0.797858 | 0.8844 |
| DLK-1 | 6.401 | 6.352 | 0.049 | 0.820417 | 0.8986 |
| LDL-receptor | 5.105 | 5.133 | -0.027 | 0.837779 | 0.9068 |
| CASP-3 | 6.525 | 6.482 | 0.043 | 0.878104 | 0.9329 |
| AZU1 | 4.068 | 4.031 | 0.037 | 0.895312 | 0.9329 |
| TFPI | 9.751 | 9.766 | -0.015 | 0.896028 | 0.9329 |
| MB | 8.87 | 8.894 | -0.024 | 0.907147 | 0.9329 |
| CHI3L1 | 7.372 | 7.346 | 0.026 | 0.912593 | 0.9329 |
| vWF | 10.031 | 10.041 | -0.009 | 0.956916 | 0.9571 |
| SPON1 | 2.839 | 2.831 | 0.008 | 0.957133 | 0.9571 |

^a^NPX units (log_2_ scale): relative quantification unit logarithmically related to protein concentration.

^b^False discovery rate by adjustment with the Benjamini-Hochberg method.

Abbreviations for the name of each proteins: refer to Table S2.

**Table S12. Independent association of differentially regulated plasma biomarkers with presence of diabetes.**

| **Biomarker** | **Mean level** | | | | | **Association with diabetic status^c^** | | | |
| --- | --- | --- | --- | --- | --- | --- | --- | --- | --- |
|  | **DM^a^ (n=27)** | **Non-DM^a^ (n=73)** | **Difference (log_2_ scale)^a^** | **Nominal  p-value** | **Adj. p-value^b^** | **Unadj. OR**  **(95% CI)** | **p-value** | **Adj. OR^d^**  **(95% CI)** | **p-value** |
| E-selectin | 12.069 | 11.552 | 0.518 | 0.0001 | 0.0115 | 6.62 (2.57-19.9) | <0.001 | 6.75 (2.37-23.1) | 0.008 |
| Interleukin-1 receptor  type 1 | 6.647 | 6.359 | 0.289 | 0.0003 | 0.0141 | 12.7 (3.09-65.6) | 0.001 | 13.4 (2.49-96.8) | 0.010 |
| Cathepsin D | 4.03 | 3.721 | 0.309 | 0.0017 | 0.0468 | 3.98 (1.53-11.5) | 0.007 | 4.63 (1.57-15.0) | 0.010 |
| Galectin-4 | 5.805 | 5.436 | 0.369 | 0.0023 | 0.0468 | 4.42 (1.75-12.6) | 0.003 | 5.19 (1.72-18.3) | 0.010 |
| Galectin-3 | 5.995 | 5.745 | 0.25 | 0.0027 | 0.0468 | 6.33 (1.79-27.7) | 0.007 | 9.72 (2.07-60.6) | 0.010 |
| Interleukin-1 receptor  type 2 | 6.931 | 6.629 | 0.303 | 0.0033 | 0.0468 | 5.05 (1.75-16.8) | 0.005 | 8.36 (2.08-41.7) | 0.010 |
| Growth differentiation  factor 15 | 6.648 | 6.132 | 0.516 | 0.0045 | 0.0468 | 2.02 (1.20-3.61) | 0.011 | 2.95 (1.26-8.13) | 0.024 |
| Intercellular adhesion  molecule 2 | 5.883 | 5.625 | 0.257 | 0.0045 | 0.0468 | 5.02 (1.61-17.6) | 0.008 | 4.05 (1.13-16.7) | 0.039 |
| Integrin beta-2 | 6.604 | 6.321 | 0.283 | 0.0046 | 0.0468 | 5.62 (1.78-20.4) | 0.005 | 8.53 (2.24-40.7) | 0.010 |

^a^NPX units (log_2_ scale): relative quantification unit logarithmically related to protein concentration.

^b^False discovery rate by adjustment with the Benjamini-Hochberg method (<5% considered significant)

^c^Per 2-fold increase in protein level.

^d^Logistic regression adjusted for age, sex, atrial fibrillation, ischemic heart disease, peak aortic velocity, and left ventricular ejection fraction.

Adj., adjusted; DM, diabetes mellitus.

**Table S13. Differentially regulated plasma biomarkers, further stratified by metformin use.**

| **Biomarker** | **Mean level** | | | **p-value**  **comparing 3 groups^b^** | **p-value**  **comparing DM groups^c^** |
| --- | --- | --- | --- | --- | --- |
|  | **Non-DM^a^ (n=73)** | **DM not on**  **metformin^a^**  **(n=10)** | **DM on**  **metformin^a^**  **(n=17)** |  |  |
| E-selectin | 11.552 | 11.977 | 12.123 | 0.0001 | 0.5317 |
| Interleukin-1 receptor type 1 | 6.359 | 6.782 | 6.568 | 0.0035 | 0.1371 |
| Cathepsin D | 3.721 | 4.069 | 4.007 | 0.0094 | 0.6701 |
| Galectin-4 | 5.436 | 5.951 | 5.718 | 0.0084 | 0.2599 |
| Galectin-3 | 5.745 | 6.17 | 5.891 | 0.0333 | 0.0928 |
| Interleukin-1 receptor type 2 | 6.629 | 6.988 | 6.898 | 0.0066 | 0.6176 |
| Growth differentiation factor 15 | 6.132 | 6.839 | 6.535 | 0.0235 | 0.3485 |
| Intercellular adhesion molecule 2 | 5.625 | 6.027 | 5.798 | 0.0300 | 0.1477 |
| Integrin beta-2 | 6.321 | 6.711 | 6.541 | 0.0153 | 0.3674 |

^a^NPX units (log_2_ scale): relative quantification unit logarithmically related to protein concentration.

^b^Comparison of biomarker levels among the non-DM, DM not on metformin, and DM on metformin groups using analysis of variance (ANOVA).

^c^Comparison of biomarker levels among the DM not on metformin and DM on metformin groups using Welch’s two-sample t-test.

DM, diabetes mellitus.

**Table S14. Functional enrichment analysis of the plasma proteome according to the presence of diabetes in patients with aortic stenosis.**

| **GO domain** | **Over-represented pathways (GO terms)** | **GO term ID** | **Adjusted p-value** | **Term size** | **Query size** | **Intersection size** | **Intersections (UniProt IDs)** |
| --- | --- | --- | --- | --- | --- | --- | --- |
| MF | Interleukin-1 receptor activity | GO:0004908 | 0.00029 | 7 | 6 | 2 | P14778, P27930 |
| MF | Interleukin-1 binding | GO:0019966 | 0.00049 | 9 | 6 | 2 | P14778, P27930 |
| MF | Carbohydrate binding | GO:0030246 | 0.00503 | 277 | 5 | 3 | P16581, P56470, P17931 |
| MF | Interleukin-1, type I, activating receptor activity | GO:0004909 | 0.03410 | 2 | 2 | 1 | P14778 |
| BP | Regulation of cellular extravasation | GO:0002691 | 0.00412 | 33 | 2 | 2 | P16581, P14778 |
| BP | Regulation of interleukin-1-mediated signaling pathway | GO:2000659 | 0.00526 | 10 | 6 | 2 | P14778, P27930 |
| BP | Cellular extravasation | GO:0045123 | 0.00560 | 69 | 9 | 3 | P16581, P14778, P05107 |
| BP | Regulation of leukocyte migration | GO:0002685 | 0.01989 | 212 | 5 | 3 | P16581, P14778, P17931 |
| BP | Neutrophil migration | GO:1990266 | 0.03112 | 122 | 9 | 3 | P14778, P17931, P05107 |
| BP | Response to interleukin-1 | GO:0070555 | 0.04227 | 217 | 6 | 3 | P16581, P14778, P27930 |
| CC | Collagen-containing extracellular matrix | GO:0062023 | 0.00084 | 421 | 7 | 4 | P07339, P56470, P17931, Q99988 |
| CC | Extracellular matrix | GO:0031012 | 0.00262 | 562 | 7 | 4 | P07339, P56470, P17931, Q99988 |
| CC | External encapsulating structure | GO:0030312 | 0.00264 | 563 | 7 | 4 | P07339, P56470, P17931, Q99988 |
| CC | Cell periphery | GO:0071944 | 0.00433 | 6178 | 9 | 9 | P16581, P14778, P07339, P56470, P17931, P27930, Q99988, P13598, P05107 |
| CC | Tertiary granule^a^ | GO:0070820 | 0.00531 | 163 | 9 | 3 | P07339, P17931, P05107 |
| CC | Ficolin-1-rich granule^b^ | GO:0101002 | 0.00761 | 184 | 9 | 3 | P07339, P17931, P05107 |
| CC | Ficolin-1-rich granule membrane | GO:0101003 | 0.03677 | 60 | 9 | 2 | P17931, P05107 |
| CC | Membrane microdomain | GO:0098857 | 0.04622 | 339 | 9 | 3 | P16581, P07339, P05107 |
| CC | Membrane raft | GO:0045121 | 0.04622 | 339 | 9 | 3 | P16581, P07339, P05107 |

^a^Secretory granule containing cathepsin and gelatinase found primarily in mature neutrophil cells; readily exocytosed upon cell activation. ^b^Highly exocytosable ficolin-1-rich, gelatinase-poor granules found in neutrophils.

GO, Gene Ontology domains; MF, molecular function; BP, biological process; CC, cellular component. UniProt IDs for proteins: refer to Table S2.

**Table S15. Functional enrichment analysis of the plasma proteome according to the presence of diabetes in patients with aortic stenosis: exploratory analysis with alternative criteria.**

| **No** | **GO domain** | **Over-represented pathways (GO terms)** | **GO term ID** | **Adjusted p-value** | **Term size** | **Query size** | **Intersection size** | **Intersections (UniProt IDs)** |
| --- | --- | --- | --- | --- | --- | --- | --- | --- |
| 1 | MF | Signaling receptor binding | GO:0005102 | 0.00008 | 1546 | 15 | 9 | P14778, P17931, Q99988, P13598, P05107, O95998, O15467, P00750, P08833 |
| 2 | MF | Interleukin-1 receptor activity | GO:0004908 | 0.00040 | 7 | 6 | 2 | P14778, P27930 |
| 3 | MF | Interleukin-1 binding | GO:0019966 | 0.00068 | 9 | 6 | 2 | P14778, P27930 |
| 4 | MF | Carbohydrate binding | GO:0030246 | 0.00634 | 275 | 5 | 3 | P16581, P56470, P17931 |
| 5 | MF | Cytokine binding | GO:0019955 | 0.00998 | 141 | 10 | 3 | P14778, P27930, O95998 |
| 6 | MF | Growth factor binding | GO:0019838 | 0.03388 | 137 | 15 | 3 | P14778, P27930, P08833 |
| 7 | MF | Signaling receptor regulator activity | GO:0030545 | 0.03940 | 548 | 11 | 4 | P17931, Q99988, O95998, O15467 |
| 8 | MF | Chemoattractant activity | GO:0042056 | 0.04348 | 36 | 11 | 2 | P17931, O15467 |
| 9 | BP | Leukocyte migration | GO:0050900 | 0.00013 | 381 | 14 | 6 | P16581, P14778, P17931, P05107, O15467, P35247 |
| 10 | BP | Myeloid leukocyte migration | GO:0097529 | 0.00037 | 227 | 14 | 5 | P14778, P17931, P05107, O15467, P35247 |
| 11 | BP | Neutrophil migration | GO:1990266 | 0.00062 | 129 | 11 | 4 | P14778, P17931, P05107, O15467 |
| 12 | BP | Response to interleukin-1 | GO:0070555 | 0.00125 | 154 | 11 | 4 | P16581, P14778, P27930, O15467 |
| 13 | BP | Granulocyte migration | GO:0097530 | 0.00132 | 156 | 11 | 4 | P14778, P17931, P05107, O15467 |
| 14 | BP | Regulation of interleukin-1-mediated signaling pathway | GO:2000659 | 0.00271 | 8 | 6 | 2 | P14778, P27930 |
| 15 | BP | Inflammatory response | GO:0006954 | 0.00418 | 756 | 13 | 6 | P16581, P14778, P27930, P05107, O15467, Q86VB7 |
| 16 | BP | Regulation of cellular extravasation | GO:0002691 | 0.00454 | 38 | 2 | 2 | P16581, P14778 |
| 17 | BP | Defense response | GO:0006952 | 0.00466 | 1687 | 14 | 8 | P16581, P14778, P17931, P27930, P05107, O15467, Q86VB7, P35247 |
| 18 | BP | Cellular response to chemical stimulus | GO:0070887 | 0.00760 | 3008 | 15 | 10 | P14778, P17931, P27930, Q99988, P05107, O95998, O15467, P00750, P35247, P08833 |
| 19 | BP | Immune system process | GO:0002376 | 0.01374 | 2748 | 11 | 8 | P16581, P14778, P07339, P17931, P27930, P05107, O95998, O15467 |
| 20 | BP | Regulation of leukocyte migration | GO:0002685 | 0.01546 | 218 | 5 | 3 | P16581, P14778, P17931 |
| 21 | BP | Cellular extravasation | GO:0045123 | 0.01651 | 72 | 2 | 2 | P16581, P14778 |
| 22 | BP | Response to external stimulus | GO:0009605 | 0.01708 | 2752 | 14 | 9 | P16581, P14778, P17931, Q99988, P05107, O95998, O15467, P00750, P35247 |
| 23 | BP | Leukocyte chemotaxis | GO:0030595 | 0.01991 | 235 | 14 | 4 | P17931, P05107, O15467, P35247 |
| 24 | BP | Response to chemical | GO:0042221 | 0.02292 | 4316 | 15 | 11 | P16581, P14778, P17931, P27930, Q99988, P05107, O95998, O15467, P00750, P35247, P08833 |
| 25 | BP | Cell migration | GO:0016477 | 0.02369 | 1454 | 14 | 7 | P16581, P14778, P17931, P05107, O15467, P00750, P35247 |
| 26 | BP | Response to stress | GO:0006950 | 0.02451 | 3780 | 14 | 10 | P16581, P14778, P17931, P27930, P05107, O95998, O15467, P00750, Q86VB7, P35247 |
| 27 | BP | Neutrophil chemotaxis | GO:0030593 | 0.02849 | 106 | 11 | 3 | P17931, P05107, O15467 |
| 28 | BP | Regulation of response to stimulus | GO:0048583 | 0.04127 | 3841 | 12 | 9 | P16581, P14778, P17931, P27930, Q99988, P05107, O95998, O15467, P00750 |
| 29 | BP | Cellular response to interleukin-1 | GO:0071347 | 0.04894 | 127 | 11 | 3 | P14778, P27930, O15467 |
| 30 | CC | Extracellular region | GO:0005576 | <0.00001 | 4303 | 15 | 14 | P16581, P14778, P07339, P56470, P17931, P27930, Q99988, P05107, O95998, O15467, P00750, Q86VB7, P35247, P08833 |
| 31 | CC | Extracellular space | GO:0005615 | 0.00012 | 3353 | 15 | 11 | P16581, P07339, P56470, P17931, Q99988, P05107, O95998, O15467, P00750, P35247, P08833 |
| 32 | CC | Collagen-containing extracellular matrix | GO:0062023 | 0.00062 | 421 | 7 | 4 | P07339, P56470, P17931, Q99988 |
| 33 | CC | Cell surface | GO:0009986 | 0.00094 | 908 | 13 | 6 | P16581, P14778, P17931, P05107, P00750, Q86VB7 |
| 34 | CC | Cell periphery | GO:0071944 | 0.00164 | 6181 | 9 | 9 | P16581, P14778, P07339, P56470, P17931, P27930, Q99988, P13598, P05107 |
| 35 | CC | Extracellular matrix | GO:0031012 | 0.00191 | 560 | 7 | 4 | P07339, P56470, P17931, Q99988 |
| 36 | CC | External encapsulating structure | GO:0030312 | 0.00193 | 561 | 7 | 4 | P07339, P56470, P17931, Q99988 |
| 37 | CC | Tertiary granule | GO:0070820 | 0.00448 | 163 | 9 | 3 | P07339, P17931, P05107 |
| 38 | CC | Ficolin-1-rich granule | GO:0101002 | 0.00643 | 184 | 9 | 3 | P07339, P17931, P05107 |
| 39 | CC | External side of plasma membrane | GO:0009897 | 0.01080 | 414 | 13 | 4 | P16581, P14778, P05107, Q86VB7 |
| 40 | CC | Ficolin-1-rich granule membrane | GO:0101003 | 0.03535 | 60 | 9 | 2 | P17931, P05107 |
| 41 | CC | Membrane raft | GO:0045121 | 0.03622 | 330 | 9 | 3 | P16581, P07339, P05107 |
| 42 | CC | Membrane microdomain | GO:0098857 | 0.03654 | 331 | 9 | 3 | P16581, P07339, P05107 |

GO, Gene Ontology domains; MF, molecular function; BP, biological process; CC, cellular component.

UniProt IDs for proteins: refer to Table S2.

**Table S16. Number of clinical events in the entire population.**

|  | **Total (n=253)** | **Non-DM (n=187)** | **DM (n=66)** | **p-value (log-rank)** |
| --- | --- | --- | --- | --- |
| Admission for heart failure | 18 (17.1) | 8 (4.3) | 10 (15.2) | <0.001 |
| All-cause death | 39 (15.4) | 23 (12.3) | 16 (24.2) | 0.009 |
| Composite of admission for heart failure and death | 53 (20.9) | 30 (16.0) | 23 (34.8) | <0.001 |

DM, diabetes mellitus.

**Table S17. Number of clinical events in the patients who underwent aortic valve replacement.**

|  | **Total (n=232)** | **Non-DM (n=172)** | **DM (n=60)** | **p-value (log-rank)^a^** |
| --- | --- | --- | --- | --- |
| Admission for heart failure | 14 (6.0) | 6 (3.5) | 8 (13.3) | 0.002 |
| All-cause death | 28 (12.1) | 16 (9.3) | 12 (20.0) | 0.030 |
| Composite of admission for heart failure and death | 38 (16.4) | 21 (12.2) | 17 (28.3) | <0.001 |

^a^Index date as the date of aortic valve replacement. DM, diabetes mellitus.

**Table S18. Predictors of unexpected admission for heart failure or all-cause mortality in the subset of patients who underwent AVR (n=232)**

|  | **Univariable** | | **Multivariable Model^a^** | |
| --- | --- | --- | --- | --- |
|  | **Crude HR** | **p-value** | **Adjusted HR** | **p-value** |
| Age (years) | 1.03 (1.01-1.09) | 0.011 | 1.03 (0.99-1.08) | 0.133 |
| Male | 1.69 (0.90-3.14) | 0.101 | 1.56 (0.83-2.95) | 0.168 |
| Diabetes | 2.97 (1.61-5.48) | 0.001 | 2.05 (1.10-3.83) | 0.024 |
| Hypertension | 1.37 (0.73-2.57) | 0.332 |  |  |
| Atrial fibrillation | 3.30 (1.71-6.35) | <0.001 | 2.65 (1.36-5.14) | 0.004 |
| Stroke | 1.15 (0.41-3.23) | 0.788 |  |  |
| Ischemic heart disease | 3.46 (1.88-6.37) | <0.001 | 2.41 (1.29-4.51) | 0.006 |
| Peak aortic velocity (m/s) | 0.63 (0.43-0.92) | 0.016 |  |  |
| LV ejection fraction (%) | 0.97 (0.95-1.00) | 0.022 | 0.98 (0.95-1.00) | 0.101 |
| TAVR (vs. SAVR) | 2.35 (0.99-5.61) | 0.054 | 1.44 (0.59-3.53) | 0.422 |

^a^ Including the same variables as the multivariable model in Table 4.

AVR, aortic valve replacement; HR, hazard ratio; LV, left ventricular; SAVR, surgical AVR; TAVR, transcatheter AVR.
